# Daily Regulation of Phototransduction, Circadian Clock, DNA Repair, and Immune Gene Expression by Heme Oxygenase in the Retina of *Drosophila*

**DOI:** 10.3390/genes10010006

**Published:** 2018-12-21

**Authors:** Milena Damulewicz, Michał Świątek, Agnieszka Łoboda, Józef Dulak, Bernadetta Bilska, Ryszard Przewłocki, Elżbieta Pyza

**Affiliations:** 1 Department of Cell Biology and Imaging, Faculty of Biology, Jagiellonian University, 30-387 Krakow, Poland; milena.damulewicz@uj.edu.pl (M.D.); bernadetta.bilska@doctoral.uj.edu.pl (B.B.); 2 Department of Biophysics, Jagiellonian University Medical College, 31-530 Krakow, Poland; mgj.swiatek@uj.edu.pl; 3 Department of Medical Biotechnology, Faculty of Biochemistry, Biophysics and Biotechnology, Jagiellonian University, 30-387 Krakow, Poland; agnieszka.loboda@uj.edu.pl (A.Ł.); jozef.dulak@uj.edu.pl (J.D.); 4 Institute of Pharmacology, Polish Academy of Sciences, 31-343 Krakow, Poland; nfprzewl@cyf-kr.edu.pl

**Keywords:** circadian rhythms, activity and sleep, neuroprotection, antimicrobial proteins

## Abstract

The daily expression of genes and the changes in gene expression after silencing the heme oxygenase (*ho*) gene were examined in the retina of *Drosophila* using microarray and SybrGreen qPCR (quantitative polymerase chain reaction) methods. The HO decrease in the morning upregulated 83 genes and downregulated 57 genes. At night, 80 genes were upregulated and 22 were downregulated. The top 20 genes downregulated after *ho* silencing in the morning modulate phototransduction, immune responses, autophagy, phagocytosis, apoptosis, the carbon monoxide (CO) response, the oxidative stress/UV response, and translation. In turn, the genes that upregulated at night were involved in translation—the response to oxidative stress, DNA damage, and phototransduction. Among the top 20 genes downregulated at night were genes involved in phototransduction, immune responses, and autophagy. For some genes, a low level of HO had an opposite effect in the morning compared to those at night. Silencing *ho* also changed the expression of circadian clock genes, while the HO decrease during the night enhanced the expression of immune system genes. The results showed that the cyclic expression of HO is important for controlling several processes in the retina, including neuroprotection and those involved in the innate immune system.

## 1. Introduction

The retina receives and transduces photic and visual stimuli. During these processes, the retina is exposed to extreme changes in light intensity and the wavelength spectrum throughout the day. During phototransduction, activated rhodopsin (metarhodopsin) is inactivated immediately after signal transduction since the accumulation of metarhodopsin-arrestin2 complexes is toxic to photoreceptors [1,2,3]. In addition, UV light and blue light exposure produces reactive oxygen species (ROS) in photoreceptors. These processes damage cells and may induce light-dependent retina degeneration [4]. However, there are mechanisms of protection against harmful short wavelength light, such as horizontal and vertical migrations of screening and photo pigments at specific times of the day [5,6]. These processes seem to be regulated by the circadian clock, which also controls other cyclic processes in the retina, such as oscillations in the amplitude of the electroretinogram (ERG) [5] and changes in the synaptic protein level [7]. 

Circadian clocks are self-sustaining generators of endogenous oscillations with a period of about a day in almost all processes in the body. The molecular mechanism of the clock in *Drosophila* is based on the circadian expression of two main clock genes, *period* (*per*) and *timeless* (*tim*). The transcripts of these genes accumulate at the end of the day and at the beginning of the night. During the night, PER and TIM proteins are synthesized. These proteins form heterodimers and enter the nucleus to repress the activity of the transcription factors clock (CLK) and cycle (CYC) [8]. CLK and CYC activate the expression of *per* and *tim*, as well as other clock genes including *vrille* (*vri*), *Par domain protein 1*ℇ (*Pdp1*ℇ), and clock-controlled genes (CCGs). When PER/TIM inhibit CLK/CYC activity, they repress their own transcription. This mechanism of the molecular clock is called the negative feedback loop. In the morning, light activates the clock photoreceptor cryptochrome (CRY) [9], and its binding to TIM targets this protein for degradation in proteasomes [10,11]. Because monomeric PER is unstable, it is also degraded. At the same time, CLK and CYC form heterodimers and bind to the E-box sequences of clock genes, activating their expression. Under light/dark (LD) conditions, the circadian clock is synchronized by light, mainly by CRY, and the period of the rhythm becomes equal to 24 h. In constant darkness (DD), the period is longer or shorter than 24 h. In addition to the central clock or pacemaker located in the brain, there are also clocks called peripheral clocks or oscillators that are distributed throughout the body. Some of these clock cells are in the retina, and they seem to be independent from the pacemaker in the brain [12], but other processes still depend on the central clock [13]. The retina circadian oscillators exhibit the circadian expression of clock genes [13,14,15,16]; however, *per* messenger ribonucleic acid (mRNA) peaks later during the day than it does in the pacemaker cells, and the PER protein is degraded sooner after the beginning of the day [16]. 

Heme oxygenase (*ho*) is one of the genes that shows circadian cycling in the retina. HO degrades heme into carbon monoxide (CO), ferrous ions, and biliverdin. In mammals, HO isoforms (HO-1 and HO-2) act as cytoprotective and anti-apoptotic agents by scavenging ROS (reviewed in [17]). In *Drosophila melanogaster*, only one form of HO, which plays a role in development [18] and in controlling the signaling pathway of DNA damage [19,20], is present. This form is also important for the protection of the retina against UV light and blue light exposure [19,21,22]. The level of *ho* mRNA peaks at the beginning of the day and during the night, and the peak in the morning decreases the light-dependent DNA damage to photoreceptors [19,21]. In arrhythmic *per*^01^ mutants, exposure to UV light increases the mortality of the flies, while *ho* over-expression decreases DNA breaks in the retina photoreceptors. Although it is clear that the morning peak in HO protects the visual system against degeneration, the role of the second peak of HO in the middle of the night is still unknown.

To understand the cellular processes regulated in the retina by *ho* in the morning (during the morning peak of motor activity) and, possibly, a new role of HO in the middle of the night during sleep, we carried out microarray analyses of isolated retinas. The changes in the expression of many genes observed after *ho* silencing indicate the importance of HO in various processes, including phototransduction, immune response, and autophagy.

## 2. Materials and Methods

### Animals and Procedures

We used the following strains of *D. melanogaster*: GMR-Gal4 (a strain that expresses GAL4 under the control of the Glass Multiple Reporter (GMR) enhancer-promoter, predominantly in the retina), UAS-*hoRNAi* (a strain that expresses dsRNA for the *ho* gene under the control of the UAS sequence) [18], and UAS-*Valium10* (a control for *hoRNAi* flies) [23]. After crossing GMR-Gal4 with UAS-*hoRNAi* or with UAS-*Valium10*, the offspring of GMR>*hoRNAi*, with *ho* expression silenced in the retina, were used as experimental flies. Those of GMR>*Valium10* with an empty *Valium10* vector expressed in the retina were used as controls. Flies were maintained under 12 h light and 12 h dark (LD12:12) conditions at a constant temperature of 24 °C. The UAS-*hoRNAi* system was used because the total knock-out of *ho* is lethal [18]. Partial *ho* silencing in the eye is sufficient to see the effect of HO on processes in the visual system [21]. Although *ho* silencing in the eye is more effective at higher temperatures, this condition causes severe retina degeneration [18].

Males, 5–7 days old, were sacrificed at the beginning of the day at ZT1 (one hour after lights-on) and in the middle of the night at ZT16 (four hours after lights-off). In total, 30 males were used for a single sample. The heads were kept in cold acetone at −80 °C for 7 days to lyophilize the tissues, and then the retinas were isolated manually. The total RNA was isolated using the Macherey Nagel XS kit (Macherey Nagel, Germany). The experiment was repeated four times. The RNA quality was controlled using an Agilent Bioanalyzer System, and the samples were sent to the OakLabs company (Berlin, Germany) for microarray data analysis on 8x60K ArrayXS *Drosophila*.

The expression of selected genes at ZT1 and ZT16 was also examined using a quantitative PCR (qPCR) technique. The total RNA was isolated using a Macherey Nagel XS kit, and the RNA quality was assessed by Nanodrop (samples used for the experiment showed A260/A280 ≥ 2.0 and A260/A230 ≥ 1.8). Of the total RNA, 1 μg was reverse-transcribed with the High Capacity cDNA Reverse Transcription Kit (Thermo Fisher Scientific, Cape Town, South Africa) and random primers. cDNA diluted 1:10 was used for SybrGreen qPCR (KapaBiosystems, Vilnius, Lithuania). The specific primers (the specificity was controlled with Primer BLAST and gel electrophoresis) used for the reaction are listed in Table 1. A standard curve was used to calculate gene expression level. The r*pl32* gene served as a reference for gene expression. Statistical analyses were initially performed using the non-parametric ANOVA Kruskal-Wallis test, followed by Tukey’s test. Statistica 7.0 software and GraphPad software were used for the analyses.

## 3. Data Analysis

We compared the data from the control flies GMR>*Valium10* collected at ZT1 and ZT16 to investigate the daily changes of gene expression in the retina. Those from GMR>*hoRNAi* with a decreased level of HO were also collected at ZT1 and ZT16 to investigate the role of HO in the regulation of genes involved in various processes in the retina in the morning and in the middle of the night.

The gene signal intensity values in each data sample have been derived from microarray studies (ArrayXS *Drosophila* Oak Labs experimentally validated the microarray based on Agilent technology). The Bioanalyzer kit “Eukaryote Total RNA Pico” was used for quality control of the total RNA prior to sample processing. The control probe signals were removed from the raw data, and the means of signals from replicate probes and signals from the same target probes were calculated. The data were then normalized according to the quantile normalization scheme and subsequently log-transformed. A two-sample *t*-test with unequal variances (Welch’s *t*-test) was used to statistically identify significantly differentially expressed genes (DEGs). Changes in gene expression values were tested using the following criteria: data from control flies vs. flies with a decreased HO level, both collected at ZT1; data from control flies vs. flies with a decreased HO level, both collected at ZT16; data from control flies collected at ZT1 vs. data from control flies collected at ZT16; and data from flies with a decreased HO level collected at ZT1 vs. data from flies with a decreased HO level collected at ZT16. Four lists of DEGs corresponding to the aforementioned criteria have been created. The lists contain 348, 304, 519, and 228 elements, respectively [for *p*-values < 0.005; 19, 20, 77, and 55 after applying a Benjamini-Hochberg’s False Discovery Rate (FDR) correction]. To ensure list robustness, ANOVA (with time and treatment as factors) was also performed on the data set. The results were partially aligned with the Welch’s *t*-test results for the statistical significance of DEGs.

The log-transformed quantile-normalized data were used to create plots showing up- and downregulated genes, which were selected according to their involvement in the biological processes of interest. 

Heatmaps showing DEGs between the control and treatment samples at ZT1 and ZT16 have also been created to provide an overall picture. 

The lists of DEGs were submitted to the online system PANTHER10 (http://www.pantherdb.org/) to perform a statistical overrepresentation test based on the Gene Ontology (GO) classification [24]. The method includes calculating the ratio of all genes in a given GO term to all genes in the background pool (the genome of *D. melanogaster* in this instance). The ratio can be used together with the binomial distribution for the null hypothesis to calculate a *p*-value parameter that describes the chance of genes appearing randomly in the results. A lower *p*-value means a lesser chance of random appearance, which means that the most significant results are those with the lowest *p*-values. The Bonferroni correction was applied to the *p*-values. Additionally, we compared our results with those presented in a set of papers by other authors [25,26,27] to confirm that our control samples showed consistent patterns of expression relative to previously published results as references for circadian expression and to determine the influence of hoRNAi-related modifications on the expression of particular genes.

## 4. Results

### 4.1. Day-Night Differences in Gene Expression in the Retina of Drosophila

Microarray data analysis showed that approximately 435 genes were differentially expressed between ZT1 and ZT16 in the control flies (GMR>*Valium10*) in the retina (*p*-value < 0.05; 112 for *p*-value < 0.005; 77 after applying Benjamini-Hochberg’s FDR correction). From these genes, we identified clock genes [*per*, *clock* (*clk*), *tim*, *vri*, *Pdp1*], known clock-controlled genes (*ebony*, *trpl*) [25,27] (Table S1) and genes regulating other processes that may also be regulated by light or that may be clock-controlled (Table S2). The highest differences in expression levels between ZT1 and ZT16 (top 20) were observed for the phototransduction genes [*trp*, *retinal degeneration A* (*rdgA*), *norpA*], DNA damage protection genes [*eukaryotic translation initiation factor 4A* (*eIF4a*), *Heat shock protein 83* (*Hsp83*), *Calmodulin* (*Cam*)] and immune system-related genes [*Neuropeptide-like precursor 2* (*Nplp2*), *Phosphoreceptor dehydrogenase* (*Pdh*)] (Table S3). Analyses using PANTHER10 showed that the most important changes in GO categories were in the nucleosome assembly, the phototransduction pathway, and the G protein signaling (Table S4).

### 4.2. Effects of Ho Silencing on Gene Expression in the Retina of Drosophila

We analyzed differences in gene expression between the control GMR > *Valium10* flies and flies with silenced *ho* expression in the retina at two different time points (ZT1 and ZT16). The microarray analysis did not show significant downregulation of the *ho* mRNA level. However, using the qPCR method, we observed a 52% decrease in *ho* expression in the GMR>*hoRNAi* strain compared to that in the control (data not shown). In this analysis, 83 genes were identified as upregulated and 57 genes as downregulated at ZT1 (Table 2). 

According to the GO categories, the most affected genes are involved in the processes of light perception, locomotion, responses to external stimuli, and geotaxis (Table 3). Moreover, among the top 20 genes upregulated in GMR>*hoRNAi* flies in the morning, we detected five genes that modulate translation, two genes that regulate transcription, four genes connected to immune responses and DNA damage, and one gene related to phototransduction (Table 3). Among the top 20 genes downregulated after *ho* silencing at ZT1 were genes responsible for phototransduction, immune responses, autophagy, phagocytosis, apoptosis, the CO response, the oxidative stress/UV response, and translation (Table 4).

In the retina of *hoRNAi* flies at ZT16, the expression of 80 genes was upregulated and 22 genes were downregulated in comparison with *Valium10* flies (Table 2). PANTHER analysis showed that the most affected processes were immune responses and oxido-reduction processes (Table 5). Most of the upregulated genes during the night in GMR>*hoRNAi* flies were involved in translation, response to oxidative stress, DNA damage, and phototransduction (Table 6). Among the top 20 genes downregulated at ZT16 were genes involved in phototransduction, immune responses, and autophagy (Table 6). Interestingly, we found that HO had opposite effects on the same gene expression at different times of the day. For example, *retinophilin* (*rtp*), *transient receptor potential* (*trp*), and *Turandot A* (*TotA*) were strongly upregulated at ZT16 and downregulated at ZT1 (Table 4 vs. Table 6).

### 4.3. Confirmation of Microarray Data with the Real-Time PCR Method

**Figure 1:**
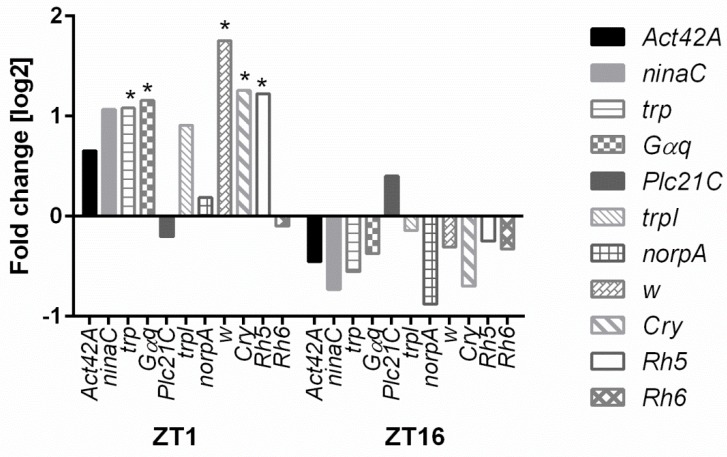
Microarray data of the phototransduction pathway genes. Differences in the selected gene expression levels are shown between GMR>*hoRNAi* and GMR>*Valium10* (control) at ZT1 and ZT16. The following genes were examined: *Act42A, ninaC, trp, Gαq, PLC21C, trpl, norpA, w*, *Crystalline* (*Cry*), *Rh5*, and *Rh6*. Statistically significant differences are marked with asterisks, * *p* ≤ 0.05.

**Figure 2:**
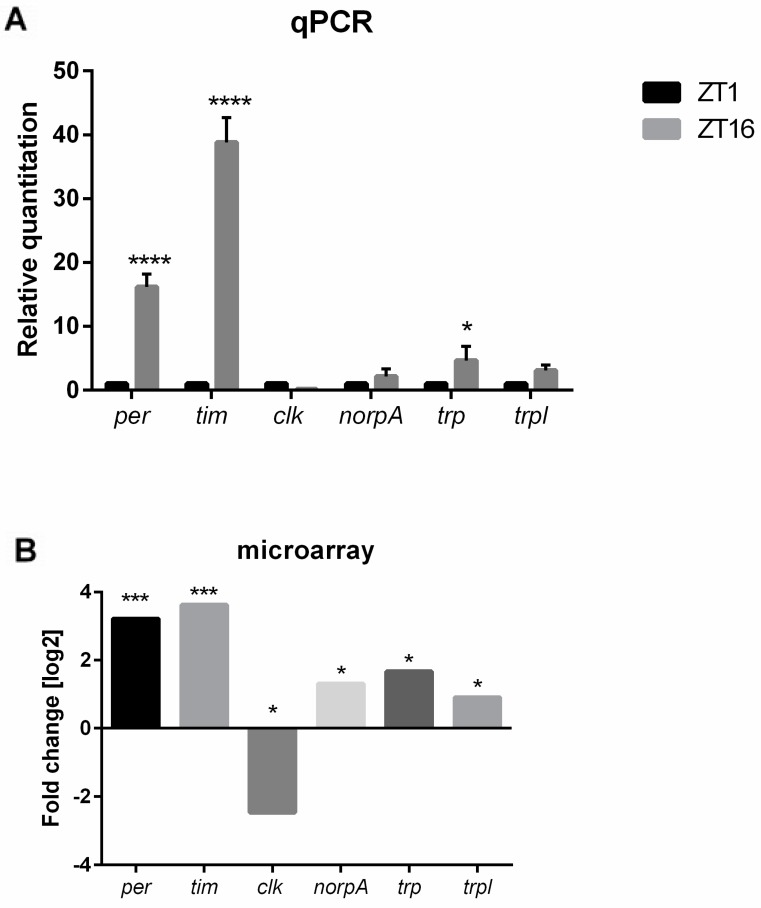
The comparison of microarray and qPCR data in control flies (GMR>*Valium10*) between ZT1 and ZT16. (**A**) qPCR data normalized to ZT1 as 1, statistically significant differences are marked with asterisks: * *p* ≤ 0.05, **** *p* ≤ 0.0001; (**B**) in microarray, fold change is relative to ZT1. Statistically significant changes are marked with asterisks: * 0.005 < *p*-values < 0.05, *** *p*-values < 0.0005. The pattern of gene expression, which was examined using two different techniques, is similar for the following genes: *per, tim, clk, norpA, trp,* and *trpl*.

In the next step of this study, we further explored the expression of genes involved in the phototransduction pathway. Microarray analyses showed that HO affects the expression of the following genes: *white* (*w*), *Crystallin* (*Cry*), *Rhodopsin 5* (*Rh5*), *Rhodopsin 6* (*Rh6*), *no receptor potential A* (*norpA*), *neither inactivation nor afterpotential C* (*ninaC*), and *trp* (Figure 1 and Figure 2). Most of these genes in GMR>*hoRNAi* flies were upregulated at ZT1 but showed no significant changes at ZT16.

**Figure 3:**
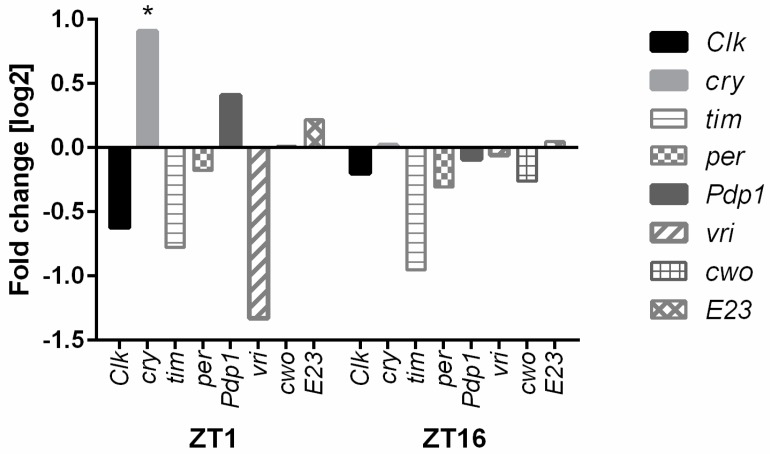
Microarray data of the circadian clock gene expression. Differences in the selected gene expression levels are shown between GMR>*hoRNAi* and GMR>*Valium10* (control) at ZT1 and ZT16. The following genes were examined: *clk*, *cry*, *tim*, *per, Pdp1, vri, cwo,* and *E23.* Fold change is relative to the control. Statistically significant difference are marked with asterisks: * 0.005 < *p*-values < 0.05.

**Figure 4:**
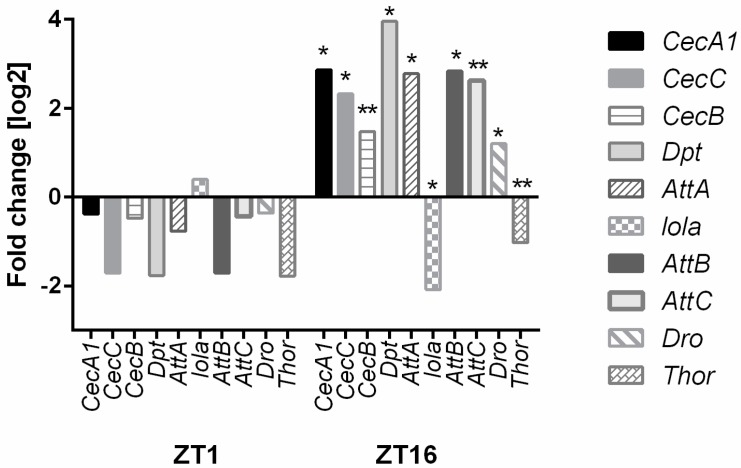
Microarray data of the humoral immune response genes. Differences in the selected gene expression levels are shown between GMR>*hoRNAi* and GMR>*Valium10* (control) at ZT1 and ZT16. Fold change is relative to the control. Statistically significant differences are marked with asterisks: * 0.005 < *p*-values < 0.05, ** 0.0005 < *p*-values < 0.005. The following genes were examined: *CecA1, CecC, CecB, Dpt, AttaA, lola, AttB, AttC, Dro,* and *Thor*.

**Figure 5:**
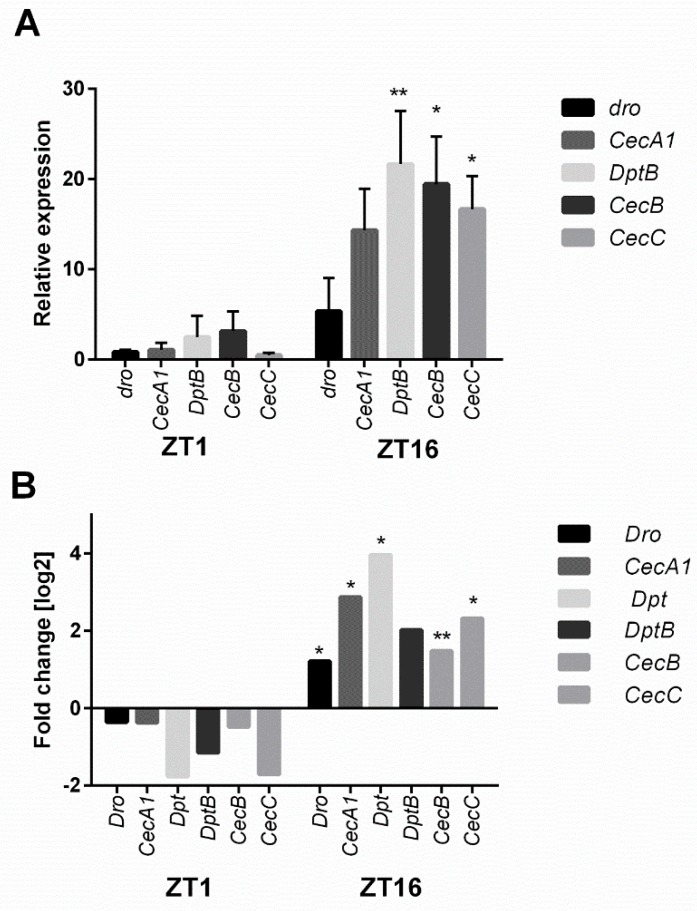
The comparison of microarray and qPCR data between GMR>*hoRNAi* and GMR>*Valium10* flies of humoral immune response gene expression levels. (**A**) qPCR data are shown as a relative quantitation normalized to the control (GMR>*Valium10*) as value 1. Statistically significant differences are marked with asterisks: * *p* ≤ 0.05, ** *p* ≤ 0.005; (**B**) in microarray, fold change is relative to the control. Statistically significant differences are marked with asterisks: * 0.005 < *p*-values < 0.05, ** 0.0005 < *p*-values < 0.005. The following genes were examined: *dro, CecA1, DptB, CecB,* and *CecC*.

Another process highly affected by *ho* silencing is the circadian clock. Microarray data showed that HO decreases the expression of *Early gene at 23* (*E23*), *cry*, and *Pdp1* in the morning and enhances *tim*, *per*, *vri*, and *clockwork orange* (*cwo*) during the night. We could see the opposite expression pattern in hoRNAi flies in the retina (Figure 2 and Figure 3). HO also strongly affects the humoral immune response, especially during the night, when the decrease in *ho* expression enhances the expression level of many genes of the innate immune system [*Cecropin A1* (*CecA1*), *Cecropin C* (*CecC*), *Cecropin B* (*CecB*), *Diptericin* (*Dpt*), *Attacin A* (*AttA*), *Attacin B* (*AttB*), *Attacin C* (*AttC*), and *Drosomycin* (*Dro*)] (Figure 4 and Figure 5).

**Figure 6:**
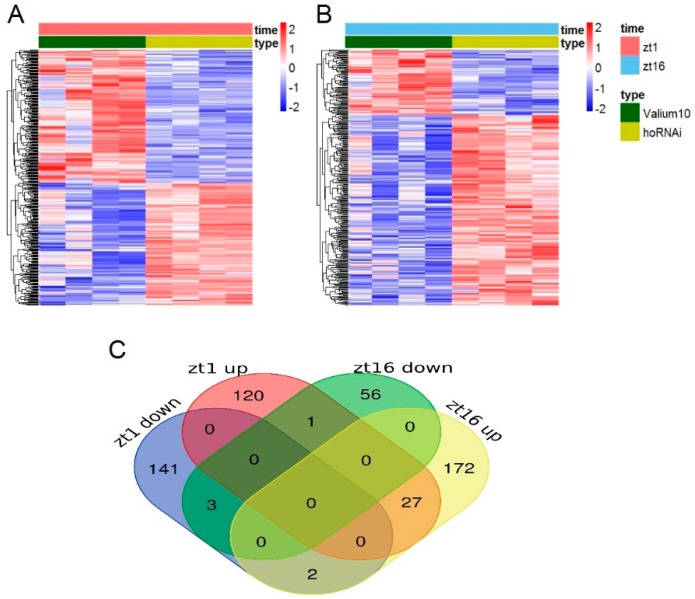
The differentially expressed genes (DEGs) between GMR>*Valium10* control samples and GMR>*hoRNAi* samples with the silenced *ho* gene (*p*-value < 0.05, fold change (FC) > 1 or FC < −1). (**A**) a heatmap showing log2 expression values of the 348 transcripts at the beginning of the day (ZT1). Each row represents one gene transcript. Values within a row were normalized and a row mean equals 0. Rows were hierarchically clustered. The 180 transcripts were downregulated while the 168 transcripts were upregulated. This allows the conclusion that the silencing of the *ho* gene leads to differences in the activity of the retina cells in the presence of light during the day; (**B**) a heatmap showing log2 expression values of the 304 transcripts in the middle of the night (ZT16). The details of the data preparation and presentation are the same as in (**A**). The 77 transcripts were downregulated while the 227 transcripts were upregulated. The analysis showed that silencing of the *ho* gene also caused changes in the retina cell activity in the absence of light during the night. The Venn diagram (**C**), which visualizes the numbers of common members of both group transcripts, clearly shows that the majority of genes responding to the *ho* gene silencing at ZT1 were not receptive at ZT16 (and vice versa). The Venn diagram takes into consideration only transcripts, which were successfully annotated (298 out of 348 in case of **A** and 263 out of 304 in case of **B**).

When we compared the DEGs between the control samples and samples with the silenced *ho* gene, we found that 180 transcripts were downregulated while 168 were upregulated, which indicates that the silencing of the *ho* gene significantly affects the activity of the retina cells at the beginning of the day (Figure 6A). In turn, among the 304 transcripts analyzed in the samples from the middle of the night (ZT16), 77 transcripts were downregulated while 227 were upregulated after *ho* gene silencing. The analysis showed that the silencing of the *ho* gene also caused a change in the retina cell activity in the absence of light during the night. Moreover, the majority of genes responding to *ho* gene silencing at ZT1 were not affected at ZT16 (and vice versa) (Figure 6B). 

In the case of the transcripts that were upregulated in the control samples at the beginning of the day, most were downregulated in the middle of the night across the relevant samples, which suggests light-dependence and/or circadian control (Figure 6). However, in part of the group of transcripts downregulated in the control samples at the beginning of the day, the expression values in the middle of the night were upregulated across all relevant samples. This suggests that these genes are normally active during the night, while, for another part of this group, the regulation patterns were similar between the beginning of the day and the middle of the night. The changes in expression caused by *ho* silencing seem to be prevalent, regardless of daylight presence or the time of day.

## 5. Discussion

In the present study, we address the role of HO in the retina of *Drosophila* at two different time points: at the beginning of the day (ZT1, high motor activity) and in the middle of the night (ZT16, sleep), when two peaks of *ho* expression were observed in our earlier study [21]. We also examined the differences among all retina gene expression levels between ZT1 and ZT16. The obtained results showed that many transcripts were upregulated or downregulated at the selected time points, and this seemed to be correlated with the high activity of the flies (ZT1) or sleep (ZT16), in addition to direct light effects at ZT1 and regulation by the circadian clock.

It has been shown that in the whole head, more than a hundred genes oscillate in a circadian manner [25,26,27,28,29,30]. However, the whole-head homogenates that were examined included brains with optic lobes, fat body, and other head tissues. In our previous study, we found that various tissues—even different parts of the visual system such as the retina and lamina—have different and specific gene expression patterns [13]. In the present study, we found that 436 genes were differentially expressed at ZT1 compared to those at ZT16 in the fly retina. Among these, 50 genes have been listed as cyclically expressed genes in whole-head homogenates [25,26,27]. These include clock genes (*per*, *tim*, *clk*) and some previously described clock-controlled genes (for example, *ebony*) [25,27]. Moreover, some of the genes that oscillate in the whole head (like *CG18386*) do not cycle in clock neurons [31], but they show daily expression changes in the retina. It is possible that other genes identified in the retina may also exhibit daily changes in mRNA levels that are light or clock-dependent, but more detailed studies are needed to examine their expression during the day.

The differences among the retinas collected at two times, ZT1 and ZT16, were highest in the genes involved in the phototransduction pathway and circadian clock. The retina photoreceptors possess self-sustained peripheral circadian oscillators [12,14] that are independent from the pacemaker located in the brain. However, some clock-controlled genes in the retina are regulated by pacemaker cells [13]. In our previous study, using a qPCR technique, we detected the cyclic expression of clock genes in the retina [13], and this is consistent with the results obtained in the present microarray data. In the case of the phototransduction pathway, it has been reported that *Rh1*, *Arr1*, and *Arr2* transcripts do not cycle [32], while *trpl*, which encodes the Ca2+ channel, has a rhythmic expression with a maximum at ZT11 [25]. We confirmed in the present study that *trp* mRNA oscillates during the day. TRP is a light-activated ion channel protein [33], and opening this channel produces light-sensitive conductance in photoreceptors. Higher *trp* and *trpl* expression during the night may increase the retina sensitivity to light. Among the phototransduction genes, *Rh5* and *Rh6* show changes in expression during the day. Claridge-Chang et al. [25] found that *Rh4* also cycles in terms of expression, but this effect was not confirmed in our study. Interestingly, the highest expression levels of *Rh5* and *Rh6* were observed at the end of the night, when the retina is most sensitive to light and when the amplitude of the ERG [5] and optomotor responses [34] are intensified. The phototransduction pathway proteins seem to be important for circadian clock entrainment since *trp/trpl* double mutants are not only blind but also have abnormal TIM degradation in response to light [35], which affects synchronization of the clock by light.

Studies performed on vertebrate cells clearly indicate that HO-1 can not only localize in cytoplasm but also be cleaved and translocated to the nucleus, where it mediates the upregulation of genes involved in the antioxidant response and cytoprotection (reviewed in [36]). This may suggest that HO-1 can act as a transcription factor, but the structure of HO-1 does not reveal traditional DNA binding motifs, which are characteristic for most transcription factors. However, interactions with other proteins in the nucleus were shown. The nuclear form of HO-1 might be a transcription factor activator or repressor, as it influences several transcription factors, thus upregulating the activity of AP-1, AP-2, STATs, and Nrf2 and downregulating NFκB or SP1 [37].

In our previous study, we found that *ho* expression and HO activity affect the molecular mechanism of the circadian clock by changing *clk* and *per* expression levels [21]. In this study, we detected that many other genes are modulated by *ho* silencing in the retina photoreceptors. Moreover, this effect is time-dependent, and the expression of the same gene changes throughout the day, as in the case of *Arr1*, *TotA*, and *rtp*. It was previously shown that *Arr1* is involved in metarhodopsin inactivation, and that changes in its location from the cytoplasm to the rhabdomere occur in a light-dependent manner [38]. *TotA* is a humoral factor secreted from the fat body under different stress factors, such as bacterial infection, high temperatures, mechanical pressure, dehydration, UV irradiation, and oxidative agents [39]. In turn, RTP is involved in organizing rhabdomeric components to suppress the random activation of the phototransduction cascade. The RTP function is also light-dependent [40]. 

In the morning, during the first peak of HO in the retina, the most important changes after *ho* silencing were observed in genes connected to the response to light. The primary role of HO at this time seems to be the protection of DNA against light-induced damage [19,20,21]. Since the phototransduction cascade enhances oxidative stress in photoreceptors [4], the observed HO-dependent decrease in phototransduction protein levels in the morning might be an additional mechanism of protection against ROS.

The phototransduction pathway is regulated by circadian inputs and HO levels. However, not all genes encoding phototransduction proteins oscillate in expression. Hartman et al. [32] reported that the most common gene in the compound eye, rhodopsin *Rh1*, is not rhythmically expressed [32], which is consistent with our data. In contrast, *Rh5* and *Rh6* expression levels are high during the night, and their mRNA levels depend on HO. *Rhodopsin 5* is sensitive to blue light and UV-A, while *Rh6* is sensitive to green light. Both are located in R8 photoreceptors, which terminate in the medulla. When HO in the retina is low, the *Rh5* and *Rh6* protein levels are higher than normal at the end of the night and at the beginning of the day. Because *Rh5* is sensitive to blue light, which is intense during the dawn and may cause retina degeneration, a high level of HO protects the eyes against ROS and DNA damage by diminishing sensitivity to blue light before dawn. At the beginning of the day (ZT1), however, HO increases the *Rh5* level and decreases the *Rh6* level. A higher *Rh5* level during the day probably allows the detection of a lower level of blue light in the environment than in the morning. At night (ZT16), *ho* silencing causes strong upregulation of the expression of chaperons (Hsc70-4), genes involved in the response to UV (*TotA*) or white light [the exit protein of rhodopsin and TRP A (*Xport*)], and DNA repair genes (*eIF-4a, Cam*) [41]. These genes seem to be involved in the repairing mechanisms of light-induced cell damage after dusk that are regulated by HO. 

During the night, HO seems to also play a role in the regulation of the immune system and redox processes, which are involved in detoxification. It has been reported that in the whole organism, antimicrobial defense is rhythmic and occurs mainly during resting time [28,42]. In turn, infections disrupt sleep efficiency and affect the circadian clock [43]. In the present study, we observed that interactions between the circadian clock and immune system also exist in the retina, and HO plays an important role in the regulation of immune response intensity. After *ho* silencing, the expression of many genes encoding antimicrobial proteins was upregulated during the night. A similar effect has been described in mammals, in which HO has an anti-inflammatory effect [17,22]. Low HO levels in the retina of our experimental flies probably increased the ROS level, and, in effect, it over-activated the immune response. A relationship between ROS and immune responses has already been detected in the gut [44]. This process in the retina is clock-dependent, and high levels of HO during the night under normal physiological conditions may also protect retina cells against overactivity of the immune system.

In summary, our data indicate that HO is an important enzyme with multiple functions in the retina that change during the day. In the morning, HO is involved in protection against UV and high ROS levels. During the night, it regulates DNA repair processes and controls the immune response intensity, playing an anti-inflammatory role in the visual system.

## Figures and Tables

**Table 1:** Primer sequences used for quantitative PCR (qPCR) reaction.

*cecB*_For	CTTCGTCTTTGTGGCACTCATC
*cecB*_Rev	CTGACCAATGCGTTCGATTTT
*dptB*_For	TCCTGATCCCCGAGAGATTG
*dptB*_Rev	CAGATCGAATCCTTGCTTTGG
*cecA1*_For	GACCTCACTGCAATATCAATATCTTT
*cecA1*_Rev	GGTGATGGCCAGAATGAGAG
*dro*_For	ATTTGTCCACCACTCCAAGC
*dro*_Rev	GATGACTTCTCCGCGGTATG
*cecC*_For	CATCAGTCGCTCAGTTTCCA
*cecC*_Rev	TTCCCAGTCCTTGAATGGTT
*trp_*For	GCCCACCGAAATAACTACGA
*trp*_Rev	TATGCGTTGATCCTCGACTG
*trpl*_For	CCAAGAAACGAAAGGAGCAG
*trpl*_Rev	GAAACATCCATTCCGCTGTT
*per*_For	AAGAGCACCTTCTGCGTGAT
*per*_Rev	AGAATCTCGTCGGGAACCTT
*norpA*_For	CCACAACATGTCCTCGTTTG
*norpA*_Rev	TGGTTGAGTTGCATGGGTAA
*rpl32*_For	AGAAGCGCAAGGAGATTGTC
*rpl32*_Rev	ATGGTGCTGCTATCCCAATC

**Table 2:** List of genes up—and downregulated after heme oxygenase (*ho) RNAi* expression under the Glass Multiple Reporter (GMR) enhancer-promoter.

Biological Process	ZT1		ZT16	
	Upregulation	Downregulation	Upregulation	Down-Regulation
**Sensory Perception**	*Gr94a* *Ir92a* CG12645 *Lush* *Gr5a* *Obp69a*		*Obp83b* *Obp84a* *Obp28a* *Or49b* *Obp69a* *Gk*	
**Transport**	*Porin2* *ppk31* CG7458 *Ndae1* CG30354 CG30345 *Ykt6* CG5780	CG1208 CG42260 *spir* *Mppe*	CG6125 CG15096 CG8051 *Mdr50* *inaF-C* *ppk22*	
**Ubiquitination**	*Roc1b*			
**Defense**	*Drs* *Rel* CG10764	*Gb*	*AttD* *Dpt* *CecC* *Thor* *AttB* *AttA* *AttC* *CecA1*	
**Translation**	CG6094 CG10881			
**Transcription**	*Hb* *Ubx* CG9727 *Vsx2* *Sug* *H15*	CG18599 *Samuel*	*lin-28* *sug* *Crg-1*	
**Metabolic Processes**	CG17544 *Oys*	CG6432 CG11453	CG11391 *Pepck*	
**Oxido-reduction**	CG10639 CG15864 CG9150	*Fbp2* CG6852 *Cyp305a1* CG5653	*Prx2540-2* *Cyp4e3* CG9629 CG10131 *Sardh* *Cyp6a14* CG10512	CG9747 *Fmo-2* CG7724
**Wound Healing**		*Cht5*		*Cht5*
**Ca^2+^ Binding**	CG30378 *TpnC47D*	CG4495		
**Cytoskeleton**	*Ppcs*	*Sls* *Mwh* *Unc-115a*	CG5023	
**Lipase**		CG1986 CG10116		CG10116
**DNA Damage**		*Corp*	*Pdk1* *mus308*	
**Proteolysis**	CG30287	CG17571 CG5255 CG4914		CG5849 CG4914
**Cuticle** **Metabolism**	CG42494 *Cpr67B*	*Cpr92F* CG15754 *Cpr5C*		
**Endopeptidase Inhibitor**				CG42464
**Response to Stress**				CG11498
**Cell Cycle**	*CycB3* *ana2*		*ana2* *Spc25* *dgt3* *Incenp*	
**Signal Transduction**	*5-HT2B* *Graf* *dlg1* *hbs* *RhoGAP100F*	*Tsp26A*	*TyrRII* *LKR* *CCAP*	*Oamb* *Proc-R*
**Metal Ion Binding**		CG42249	CG18446 *MtnD* *Amy-p*	
**Phosphatase**		*Aph-4*	CG11425	*Aph-4*
**Hydrolase**			CG7997 *Tobi*	*Jhe*
**ATPase**	CG9492	CG11069		
**Kinase**	*CkIIalpha-i3* CG15547			
**Transferase**	CG3038			
**Nucleic Acid Binding**	CG3165	CG11563 CG7556 Adat	*cid* CG45050 *qkr58E-1*	
**Lipid Binding**	*CheB42b*			
**Phagocytosis**	*Prx3* *NimA*		*Prx3*	
**Zinc Binding**	CG31053 *RAF2*	*CAH1* *App*		
**Phototransduction**	*Rh5*			
**Geotaxis**	CG34353			
**Clock**		*Cry*		
**Response to ER Stress**			CG42678	
**Septate Junction**			*Tsp2A*	
**Unknown**	CG13742 CG43894 CG43391 *scpr-C* *abd-A* CG3611 CG43816 CG17564 CG15186 CG15140 CG32413 CG34279 CG13337 CG12688 CG44437 CG13449 CG4998 CG12321 CG44158 CG42869 CG11905	CG10051 CG32548 CG45060 *G6P* CG44163 CG31415 CG13058 *Osi24* CG13332 CG12034 CG43117 CG15728 *pyd3* *Sfp77F* *pre-mod(mdg4)-B* CG17777 CG42272 *scpr-B* CG45603 CG11380 CG17378	*Os-C* *α5* *CG17572* CG12239 CG11674 *OS9* *Tsp42Er* CG4962 CG30271 *a10* CG10332 *Lsp1beta* CG45061 *pre-mod(mdg4)-T* CG11585 *MESR3* CG18170 CG13042 CG14075 *Lsp1alpha* CG43236	CG13840 CG44434 CG11889 CG45307 *Sfp77F* CG17777 *scpr-B* CG17104 CG13931 CG8568

**Table 3:** List of the most changed Gene Ontology (GO) categories after *ho* silencing in the retina at ZT1.

GO Category ZT1 vs. ZT1 GMR>*hoRNAi*	Gene Count	*p*-Value	Genes
Locomotion (GO:0040011)	27	1.46 E-01	*NetA*, *RhoGAP100F*, CG34353, *Ppcs*, *Trpm*, *fra*, *oys*, *ey*, *Nmda1*, *hb*, *Roc1b*, CG3857, *Tsp26A*, *how*, *Cry*, *cry*, *abd-A*, *w*, *Galphaq*, *trp*, *Ced-12*, *dlg1*, *sls*, *stum*, *Unc-115a*, CG9317, *klar*, *E(Pc)*
Sensory Perception of Light (GO:0050953)	5	3.37 E-01	*Rh5*, *cry*, *Cry*, *trp*, CG9317
Response to External Stimuli (GO:0009605)	34	3.86 E-01	*NetA*, *RhoGAP100F*, *NimC1*, *Atg6*, CG34353, *Trpm*, *fra*, *Su(var)2-10*, CG2051, *Rh5*, *Nmda1*, *Drsl5*, *rdgB*, CG3857, *mthl8*, *trp*, *Cry*, *cry*, CG13890, *Drs*, *sug*, *w*, *Galphaq*, *Gr5a*, *dlg1*, *Diedel*, *gb*, *Sik2*, *Fuca*, *stum*, *Unc-115a*, CG9317, *E9Pc*, *Rel*, *srpk79D*
Geotaxis (GO:0042332) Response to Gravity (GO:0009629)	5	5.79 E-01	CG34353, CG3857, *Cry*, *cry*, *w*, *dlg1*

**Table 4:** Top 20 genes up- and downregulated after *ho* silencing in photoreceptor cells at ZT1.

**Genes Upregulated at ZT1** **in GMR>*hoRNAi* Strain**	**Function**
*Arr1*	Phototransduction
CG17108	Unknown
*MntA*	Metal homeostasis
*eIF-4a*	Response to DNA damage
*Mt:Cyt-b*	Electron transport
*RpL13*	Translation
*RpS25*	Translation
*RplP2*	Translation
CG3457	Unknown
*IM2*	Defense response
*RpL4*	Translation
CG4374	Metal binding
*To*	Circadian clock
*RpS27A*	Translation
CG9821	Unknown
*Trf4-1*	DNA polymerase
*Awd*	Kinase
*Spt20*	Histone acetylation
*IM4*	Defense response
*Ip259*	Phagocytosis
**Genes Downregulated at ZT1** **in GMR>*hoRNAi* Strain**	**Function**
*Obp99b*	Sensory perception
*Nplp3*	Signaling pathway
*Arr2*	Phototransduction
*ninaE*	Phototransduction
*Pdh*	Phototransduction
*Mt:ATPase6*	ATPase
*Ggamma30A*	Phototransduction
*mt:Col*	Unknown
CG2233	Unknown
*Trp*	Phototransduction
*Obp44a*	Sensory perception
*RpL27A*	Translation
*Mt:ColII*	Unknown
*Nplp2*	Defense response
*GstE12*	Detoxification
*Rtp*	Unknown
*RpS28b*	Translation
*TotA*	Response to UV, oxidative stress, bacterium
*CG4842*	Oxido-reduction
*Sea*	Prevents chromosomal breaks

**Table 5:** Top 10 of the most changed GO categories after *ho* silencing in photoreceptors at ZT16.

GO Category	Gene Count	*p*-Value	Genes
Antibacterial Humoral Response (GO:0019731)	10	8.00 E-08	*CecA1*, *CecC*, *AttA*, *Thor*, *Dpt*, *CecB*, *AttB*, *AttC*, *CecA1*, *Dro*
Antimicrobial Humoral Response (GO:0019730) Humoral Immune Response (GO: 0006959)	11	1.67 E-05 4.25 E-04	*CecA1*, *CecC*, *AttA*, *Thor*, *Dpt*, *CecB*, *AttB*, *AttC*, *CecA1*, *Dro*, *lola*
Defense Response to Gram-Positive Bacterium (GO:0050830)	9	2.29 E-04	*AttD*, *CecA1*, *CecC*, *AttA*, *CecB*, *AttB*, *AttC*, *CecA1*, *Dro*
Oxidation-Reduction Process (GO:0055114)	26	2.18 E-02	CG33099, CG9747, CG9629, *Sardh*, *Cyp4e3*, *Lkr*, CG18003, *Cyp309a1*, *Prx3*, CG10131, *MtnD*, *Acox57D-d*, *Cyp6a14*, CG10512, *Cyp9h1*, CG11236, CG18170, CG3397, CG14688, *Prx2540-2*, CG7724, *Cyp4p2*, CG14630, *Sodh-2*, *Fmo-2*, *GILT3*
Cellular Macromolecule Metabolic Process (GO:0044260)	17	5.03 E-02	CG13085, *Su(var)2-10*, CG43143, *Lkr*, *mus308*, *fng*, *qrk58E-1*, *MED23*, *S6k*, *Sse*, *Fak*, *RpS21*, *btl*, *p38c*, *Pdk1*, *Incenp*, CG18853
Single-Organism Metabolic Process (GO:0044710)	46	1.40 E-01	CG33099, CG9747, CG10184, CG9629, *Sardh*, *Cyp4e3*, *Vkor*, *Lkr*, CG18003, CG5379, *Cyp309a2*, *Prx3*, CG10131, *Thor*, CG10116, *MtnD*, *Crz*, *Acox57D-d*, *Pepck*, *Cyp6a14*, *fng*, CG10512, CG7059, CG6465, *AcCoAS*, *dob*, *Cyp9h1*, CG11236, CG18170, *S6k*, CG11425, CG3397, *Jhe*, CG6415, CG14688, *Gnmt*, *Prx2540-2*, CG7724, *Sk1*, *ade3*, *Cyp4p2*, CG14630, *Sodh-2*, *Fmo-2*, *Lsd-1*, *GILT3*
Defense Response to Other Organism (GO:0098542)	14	2.28 E-01	*AttD*, *Drsl6*, *CecA1*, *Su(var)2-10*, *CecC*, *AttA*, *Thor*, *Dpt*, *CecB*, *GNBP-like3*, *AttB*, *AttC*, *CecA1*, *Dro*
Immune Response (GO:0006955)	12	2.83 E-01	*CecA1*, *CecC*, *AttA*,*Thor*, *Dpt*, *CecB*, *AttB*, *AttC*, *PGRP-SC2*, *lola*, *Dro*
Response to Bacterium (GO:0009617)	13	3.05 E-01	*AttD*, *CecA1*, *Su(var)2-10*, *CecC*, *AttA*, *Thor*, *Dpt*, *CecB*, *AttB*, *AttC*, *CecA1*, *p38c*, *Dro*
Response to External Biotic Stimulus (GO:0043207)	16	3.07 E-01	*AttD*, *Drsl6*, *CecA1*, *Su(var)2-10*, *CecC*, *AttA*, *Thor*, *Dpt*, *CecB*, *GNBP-like3*, *AttB*, *AttC*, *CecA1*, *lola*, *p38c*, *Dro*

**Table 6:** Top 20 genes up- and downregulated after *ho* silencing in photoreceptor cells at ZT16.

**Genes Upregulated at ZT16 in GMR>*hoRNAi* Strain**	**Function**
*Rh6*	Phototransduction
*TotA*	Response to UV, oxidative stress, bacterium
*Trp*	Phototransduction
*RpS13*	Translation
*Xport*	Phototransduction
*eIF-4a*	DNA damage
*RpL37A*	Translation
CG4962	Unknown
*Rtp*	Unknown
*Ggamma30A*	Phototransduction
*Cam*	DNA damage
*RpS7*	Translation
*RpS26*	Translation
CG4000	Unknown
*Hsc70-4*	Chaperon
*RpL8*	Translation
*RpS28b*	Translation
CG1561	Unknown
CG2016	Unknown
*RpLP1*	Translation
**Genes Downregulated at ZT16 in GMR>*hoRNAi* Strain**	**Function**
*Obp99b*	Sensory perception
*Arr1*	Phototransduction
*Arr2*	Phototransduction
CG17108	Unknown
CG6503	Unknown
*Tsf1*	Defense response
*Lsp2*	Defense response
*mt:ATPase6*	ATPase
*Nplp2*	Signaling pathway
*Nplp3*	Signaling pathway
*mt:Cyt-b*	Oxido-reduction
CG2233	Unknown
*Adhr*	Oxido-reduction
*mt:Col*	Unknown
*Pepck*	Glucose metabolism
*RpLP2*	Translation
CG34166	Unknown
*mt:ND4*	Oxido-reduction
*Noe*	Unknown
CG30197	Peptidase inhibitor

## Supplementary Materials

The following are available online at http://www.mdpi.com/2073-4425/10/1/6/s1. Figure S1. Genes with different expression levels between ZT1 and ZT16 in the retina (according to our microarray data), which were described as circadian genes in the whole head of *Drosophila* by other authors. Figure S2. Genes with different expression levels between ZT1 and ZT16 in the retina (in our microarray data) but not in the whole head reported by other authors. Figure S3. Top 20 genes up- and downregulated at ZT16 in control flies. Figure S4. Top 10 genes of the most changed GO (gene ontology) between ZT1 and ZT16 in control flies. Figure S5. The differentially expressed genes across all samples (*p*-value < 0.05, FC > 1 or FC < −1). (A) a heatmap showing log2 expression values of 348 transcripts from Figure 6A with the addition of ZT16 samples. In the case of a group of transcripts that were upregulated in the control samples at the beginning of the day, most of them were downregulated in the middle of the night across relevant samples. This suggests that silencing of the *ho* gene results in decreased sensitivity to the daylight. However, in part of a group of transcripts downregulated in the control samples at the beginning of the day, the expression in the middle of the night was upregulated across all relevant samples, which suggests that these genes can be active in the absence of daylight. Whereas, for another part of this group, the regulation pattern was similar when gene expression levels were compared between the beginning of the day and the middle of the night. Changes in expression caused by the *ho* gene silencing seem to be prevalent, regardless of the daylight presence; (B) a heatmap showing log2 expression values of 304 transcripts from Figure 6B with the addition of ZT1 samples. The group of transcripts upregulated at ZT16 in the control GMR>*Valium10* samples could be either connected to processes which occur during the night, since they are not upregulated at ZT1, or engaged in long term processes of protection against short wavelength light. The group of transcripts downregulated at ZT16 in GMR>*Valium10* samples seems to have three distinctive patterns of expression. From the top, the first and largest pattern at ZT1 suggests that the difference in expression of these transcripts is driven only by silencing of the *ho* gene and is not connected to day/night cycle, even if the difference at ZT1 is not as clear as at ZT16. However, the *ho* gene expression is clock-dependent. The second pattern shows that there are some genes that are upregulated during the day in all samples, but are downregulated during the night only when the *ho* gene is not silenced. It suggests that HO is not only responsible for active defense against short wavelength light, but also involved in other functions, for example in the regulation of immune responses and DNA repair. The third pattern is related to transcripts whose expressions are reversed when gene expression is compared between two time points of both types of samples. This result indicates a possibility that *ho* silencing disturbs the daily rhythms in gene expression; (C) a heatmap showing log2 expression values of 519 transcripts differentially expressed between ZT1 and ZT16 of the GMR>*Valium10* (control) samples with the addition of GMR>*hoRNAi* samples. Aside from a relatively small group of transcripts near the middle of the heatmap, the prevalent expression pattern suggests that the difference between ZT1 and ZT16 in control samples is nullified by silencing of the *ho* gene, in agreement with our expectations of *ho* silencing effect; (D) a heatmap showing log2 expression values of the 228 transcripts differentially expressed between ZT1 and ZT16 of the GMR>*hoRNAi* samples with the addition of control samples. There are four distinctive patterns in the added control samples. From the top, the first pattern includes transcripts whose expression levels seem to be not affected by *ho* silencing. The second pattern includes transcripts whose expression levels in the added control samples were uniformly raised in comparing with their levels in ZT1 samples with the silenced *ho* gene, but similar to the levels of expression in ZT16 samples with silenced *ho*. This suggests that the decreased *ho* expression causes these genes to be suppressed only during the day and/or in the presence of light during the day. The third pattern, similar to the first one, follows what was established in the GMR>*hoRNAi* samples, which means that these genes are most likely not connected to the *ho* gene functions. The fourth pattern pertains to a small group of transcripts whose expression levels seem to be switched and, as a result, transcripts that were upregulated at ZT1 of GMR>*hoRNAi* samples were downregulated at ZT1 of the control samples (and vice versa). This phenomenon raises a possibility that *ho* gene silencing disturbs light-dependent processes and the circadian clock; (E) a Venn diagram in which the numbers of common elements between each heatmap A, B, C, and D are shown. It suggests that lists of these heatmap genes are largely independent from each other. The Venn diagram takes into consideration only transcripts which were successfully annotated (298 out of 348 in case of A, 263 out of 304 in case of B, 445 out of 519 in case of C, and 209 out of 228 in case of D). The details of all the heatmap preparations and presentations are the same as in Figure 6. Figure S6: List of genes from the heatmap A (Figure 6) with their fold change, Welch’s *t*-test *p*-values, and ANOVA *p*-values adjusted for FDR. Figure S7: List of genes from the heatmap B (Figure 6) with their fold change, Welch’s *t*-test *p*-values, and ANOVA *p*-values adjusted for FDR.
