# Supplementary material for: Daily Regulation of Phototransduction, Circadian Clock, DNA Repair, and Immune Gene Expression by Heme Oxygenase in the Retina of Drosophila
Source: Genes (Basel). 2018 Dec 21;10(1):6. doi: 10.3390/genes10010006 (PMC6357063; doi:10.3390/genes10010006)
Supplement: Supplementary file 1 [file genes-10-00006-s001.pdf]

Figure S1. Genes with different expression between ZT1 and ZT16 in the retina (according to our microarray data) which were described as circadian in whole head (according to Claridge-Chang et al., 2001; McDonald and Rosbash, 2001; Ueda et al., 2002).

Biological process	Genes
**Nucleic acid metabolism**	*vn, bel*, CG17386, CG9705
**Synaptic function**	*Slob, e, 5-HT1A,5HT2, ple*
**Sensory function**	*trpl, pn, Rh5, loco, Nsf2, Akap200, MESR3, Cnx99A*
**Transport**	CG8468, CG2121, CG6293, CG9990, CG8034, CG3823, CG10237
**Cytoskeleton**	*TpnC47D*, CG9377
**Protein cleavage**	*ea*, CG4723, CG9634, CG7288, CG9377
**Aminoacid metabolism**	CG10184, *Ahcy89E*
**Lipid metabolism**	*ATPCL*, CG10253*, Inos*, CG11425
**Oxidoreductases**	CG15093, CG12116, CG11796, CG7724, *Sodh1*
**detoxification**	*Cyp6a21, Cyp305a1, Cyp18a1, Cyp4d21*, CG8993, *Ugt35a, Cyp4d21*
**Circadian clock**	*per, clk, tim, cry, vri, Pdp1*
**hydrolase**	CG15093, CG10237
**unknown**	CG11854, CG11889, CG9497, CG10513, CG10433, CG17777, CG14275, CG4962
**Other**	*Eip55E*, CG1441, CG8036, CG6218, *Tpi*, CG2765, CG5446, CG7149

Figure S2. Genes with different expression between ZT1 and ZT16 in the retina (in our microarray data) but not in the whole head (according to Claridge-Chang et al., 2001, McDonald and Rosbash, 2001; Ueda et al., 2002).

Biological process	Upregulated at ZT16	Downregulated at ZT16
**Transport**	*Gp210, hoe2, Irk1, Bmcp, Ir68a,* CG31100, *Csp, Mfrn, w, Vha16-3, blot*, CG4607, CG6299, CG10237, CG5535, *Cpx, Prestin, Kap3, Ykt6, sea*	*Orco, Mdr49, Cngl, Shab,* CG3823, CG9657, *Best3, Ranbp16, Npc2f, Porin2,* CG6836, CG8785, CG7720, *NAAT1, Ir75a*, CG1208
**Morphogenesis**	*Dy*	
**Defence response**	*Pli*, CG2051, *Rm62,*	*AttB, srpk79D*, CG10433, *Kn*
**DNA damage**	*Gclm, Lola, Ball, Pvr*	
**Wound healing**	*Eaf, chic*	*NijA, aralar1*
**Phagocytosis**	*Act42A*	CG31064, *sr*, CG6364
**Autophagy**	*PP2A-B'*	*Br*

**Response to heat**		CG7409
**Response to oxidative stress**		IP3K1
**Transferase**	CG5196, *Oys, Dot, Alg10, l(2)not*	*GstE9*, CG17219, *fu12*, CG31776, *isoQC*
**GTPase**		CG32506
**ATPase**	*Vha36-3, Myo28B1*	CG17646
**Lipase**	CG1986, *Cnx14D, Hydr2*	*Dob*
**Kinase**	CG31145, *PI4KIIIalpha, Sik2, Sk1*	*Ddr, CycJ*, CG8414, CG15547, CG8173, *Takl2*, CG32944, *Drak*
**Phosphatase**	CG2680, CG12078	CG11597, CG11425
**Hydrolase**		CG6201, *Jhe*, CG9119
**Ubiquitination**	*Ubpy, Gol*, CG42797, CG8419, CG2681, CG9855, *ntc*, CG7656	*Roc1b*
**Cytoskeleton**	CG7261, *Dia, Ck, Ced-12*	CG32371, *Unc-115b, Cep135, Unc-115a*
**Cell adhesion**	*Chp, Rols*	
**Signal transduction**	*AdoR, Exn, uif, E(spl)m2-BFM, TyrRII, Hug, Fng, Mam*, CG5916, *Pde9*, CG4972, *boss, dlg1, Fs, Lst8, tow*	CG30456, *RhoGAP100F*, CG6405, *spz3, hbs, Pde1c, PsGEF, fz, Ac76E,Oamb, TkR99D, Pde6*, CG14669, *mthl8, RhoGEF3, mAChR-B, NT1*, CG30456, CG7497, *Mtt*
**Clock**	*vri, Pdp1, cwo,* CG2650,	*cry*
**oxidoreduction**	CG1434, *Cyp4ae1, Eip71CD, Prx2540-2, Cyp12b2*, CG10863	CG13334, *l(2)01289*, CG30354, CG7724, CG11796, *Cyp308a1*, CG10512, *RnrS*
**biosynthesis**	CG4825, *bnb*, CG17544, *Gs2, Pbgs*	CG8613, CG8343, *AdSL,* CG5065
**phototransduction**	*rdgA, Cnx99A, ninaC, trp, Cry, Rh5*, CG9317, *norpA, rdgB, ninaG, Galphaq, stops*	*Plc21C*
**transcription**	CG2652, *His1:CG33864, MED1, CTCF, tin, NAA20*, CG13188, *MED27, Onecut*, CG2926, *Pph13*	*Retn, Incenp, Dll, Hr38, C15, Samuel, FoxP, vfl, lin-28,* CG11762, *E(Pc), H15, Pnr, sage, fd102C, Gce, Cas, spn-E*
**translation**	*mRpL38*, CG5989, *l(3)07882, mRpS31, mxt,* CG12413	
**Nucleic acid binding**	CG3335, CG41562, *Phax*	
**Sensory perception**	*Obp56h*, CG14636, CG2698	*OS9, Obp69a lush*, CG18557, *Obp49a, CG1387, dpr5, Gr2a, Gk*
**Proteolysis**	*Jon99Ciii*, CG11034, CG13366, CG31954, *Lon*, CG17739, CG5909	CG4678, CG3604, CG3499
**Metal binding**	CG11825	*Glut4EF*, CG8910, CG17912
**Ca binding**	*Tsp, Edem1*	
**Zn binding**	CG6808, *Dwg, Hil,* CG12795	CG45050, CG31053
**learning**	*Nord*	
**Cuticule metabolism**	CG7017, *kkv*	*Cpr72Ec*

**Golgi organization**	CG10075	
**Pigmentation**	*Pu*	
**ATP binding**		CG14535
**Galactose binding**		*lectin-37Da*
**Metabolic**		*Vkor*, CG11453
**Synapse organization**		*Neto*
**Glia migration**		*NetA*
**Septate junction assembly**		*Tsp2A*
**apoptosis**		CG10257, *Corp*
**Cell cycle**	*EndoG*	*Rcd2, twe*
**unknown**	*Mst84Da, Cyr, Tsp42Eo, Tsp42En*, CG13705, Osi6, CG42808, CG31710, CG1636, CG42357, CG11380, CG4666, CG13272, CG17777, *thoc6*, CG40486, CG10479, CG44434, CG11307, CG14275, CG14223, CG1850, *Sgs1,* CG31688, CG4962, CG8765, CG1561, CG4367, CG13071, CG13606, CG4669, CG7906, *Pim,* CG9782, CG13063, *Nmda1*, CG43783, CG13042, CG43781, CG11585, CG2150, *defl*, CG10311, CG4982, *sowah*, CG10623, CG3501, CG31344, CG8568, CG13003, CG13563, CG32554, CG43078	*a10*, CG34180, CG2082, CG32676, CG42402, CG10407, CG17666, CG30069, CG42656, CG14142, CG10560, CG32512, CG3611, CG32023, CG18628 CG11409, CG34219, CG32793, CG34136, CG16798, CG11550, CG42764, CG13203, CG43707, CG43117, CG16959, CG7702, CG31157, CG12239, CG43095, CG10513, CG30356, CG43172, CG42368, CG34184, CG34115, CG11889, CG32040, CG1324, *2mit*, CG32553, CG34033, CG13875, CG44004, CG42343, CG32563, CG8907, CG18745, CG32066, CG8204, CG13694, CG42540, CG15544, CG32407, CG13318, CG11854, CG42675, CG43094, jb, CG33993, CG43341

Figure S3. Top 20 genes up and downregulated at ZT16 in control flies.

Genes upregulated at ZT16	Function
Rh6	Phototransduction
trp	Phototransduction
Ggamma30A	Phototransduction
**CG1561**	Unknown
Inos	Lipid metabolism
Nplp3	Neuropeptide signaling pathway
RpS13	Translation
rtp	Unknown
RpL27A	Translation
Xport	Phototransduction
Sea	Citrate transport, prevents chromosomal breaks
Obs44a	Sensory perception
GstE12	Detoxification

RpS28b	Translation
Mbl	Regulation of gene expression
Cam	DNA damage, autophagy
RpS7	Translation
RpL37A	Translation
Trpl	Phototransduction
**CG4962**	Unknown

**Genes downregulated at ZT16**	**Function**
Arr1	Phototransduction
**CG17108**	Unknown
MtnA	Metal homeostasis
mt: Cyt-b	Oxidoreduction
Arr2	Phototransduction
Pdh	Phagocytosis, phototransduction
RpLP2	Translation
**CG6503**	Unknown
eIF-4a	Splicing, DNA damage
**CG10433**	Unknown
RpL13	Translation
Mt:CoII	Unknown
RpS25	Translation
Hsp83	Chaperon
Tsf1	Response to fungus
Idh	Fatty acid oxidation
To	Clock
Noe	Unknown
Gapdh2	Oxidoreduction
Cpr72Ec	Cuticule development

Figure S4. Top 10 of the most changed GO (gene ontology) between ZT1 and ZT16 in control flies.

**GO category**	**Gene count**	**p-value**	**genes**
**Nucelosome assembly (GO:0006334)**	19	2.21E-07	*His1:*CG33864, CG2051
**Chromarin assembly (GO:31497)**	19	7.56E-07	
**Protein-DNA complex assembly (GO:0065004)**	19	2.01E-06	
**Chromatin assembly or disassembly (GO:0006333)**	19	2.98E-05	
**Nucleosome organization (GO:0034728)**	20	2.60E-06	*His1*:CG33864, CG2051, *E(Pc)*

**Protein-DNA complex subunit organization (GO:0071824)**	20	3.53E-05	
**DNA packaging (GO:0006323)**	22	6.85E-06	*His1*:CG33864, CG2051, *ball, spn-E, Incenp*
**DNA conformation change (GO:0071103)**	22	2.13E-05	
**Protein complex assembly (GO:0006461)**	24	1.10E-03	*His1*:CG33864, CG2051, *Shab, Trpm, chic, Orco,* CG7261
**Protein complex biogenesis (GO:0070271)**	24	1.27E-03	
**Protein complex subunit organization (GO:0071822)**	27	1.27E-03	*His1*:CG33864, CG2051, *Shab, Trpm, chic, Orco,* CG7261, *Csp, Gp210, E(Pc)*
**Cellular response to light stimulus (GO:0071482)**	9	3.50E-03	*TotZ, rdgB, norpA, trp, Cry, cry, Galphaq, tim, ninaC, rdgA*
**G-protein coupled receptor signaling pathway (GO:0007186)**	20	7.23E-03	*TkR99D, mtt,* CG7497, *5-HT2A, Rh5, AdoR, rdgB, norpA, mthl8, trp, Gaphaq, boss, ninaC, mAChR-B, 5-HT1A, Oamb, rdgA, fz, Hug, TyrRII*
**Detection of visible light (GO:0009584)**	8	7.81E-03	*rdgB, norpA, trp, Galphaq, ninaC, rdgA*, CG9317, *trpl*
**Phototransduction, visible light (GO:0009584)**	7	8.86E-03	*rdgB, norpA, trp, Galphaq, ninaC, rdgA, trpl*
**Rhodopsin mediated signaling pathway (GO:0016056)**	6	9.48E-03	*rdgB, norpA, trp, Galphaq, ninaC, rdgA,* CG9317

Figure S5 Differentially expressed genes across all samples

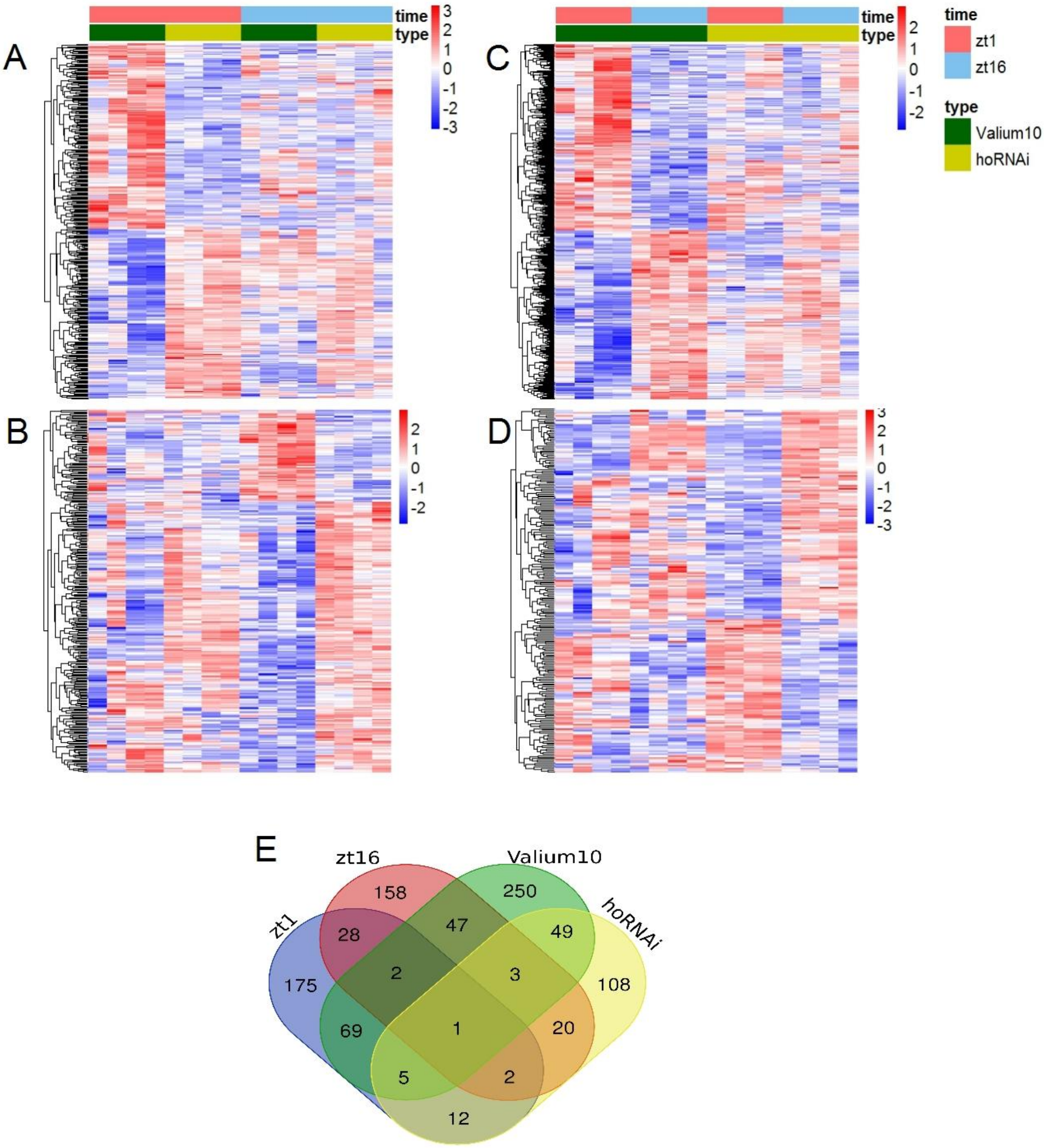
A
B
C
D
E
time
type
zt1
zt16
Valium10
hoRNAi
3
2
1
0
-1
-2
-3
zt16
Valium10
zt1
hoRNAi
158
250
28
47
49
175
2
3
108
69
1
20
5
2
12

Figure S6: List of genes from the heatmap A (Fig. 6), with their fold change, Welch's t-test p-values and ANOVA p-values adjusted for FDR.#
#

	gene names#	fold change	Welch's t-test p-value	adjusted p-value
1	CG10116	6.75698	4.5e-08	1.27825724798632e-05
2	CG13796	-4.41153	9.399e-06	0.00739315515446013
3	CheB42b	4.40526	1.2214e-05	0.339397752674255
4	Sfp77F	3.91166	1.4642e-05	0.0203261123926112
5	Porin2	3.73485	3.8465e-05	0.337096483355174
6	CG11854	3.43353	5.3937e-05	0.185075946469654
7	Spn77Bb	3.3989	5.8277e-05	0.00740922120863881
8	Unc-115a	-3.29402	5.8694e-05	8.17974187151398e-05
9	CG11380	-3.20065	6.8437e-05	0.655615077905348
10	CG17564	-3.1649	9.7399e-05	0.754345163817909
11	Mst84Da	-2.99513	0.000133496	0.757671286032475
12	scpr-B	-2.91815	0.000212395	0.000137232337333212
13	Prx3	2.85876	0.000254527	0.0130535106540621
14	aurB	2.77661	0.000303766	0.00319853323991776
15	isoQC	2.70678	0.000322335	0.373297547916074
16	CG45603	-2.56389	0.000324381	0.720259439306979
17	CG5653	-2.55953	0.000433656	0.344757674868181
18	CG9509	-2.4985	0.000494619	0.117771822149298
19	CG17597	2.46265	0.000535532	0.00080508688024098
20	CG44163	2.45967	0.000542459	0.533988060465813
21	CG1208	2.45902	0.000603402	0.125877324937865
22	Aph-4	-2.43751	0.000827451	0.00106010354186678
23	Gr2a	-2.43183	0.000910287	0.370785838588849
24	Tsp42Eb	2.39254	0.000951906	0.185075946469654
25	CycB3	-2.39028	0.000987023	0.794327317828636
26	Irk1	-2.34888	0.00118974	0.856832143139358
27	CG11034	-2.34723	0.00134142	0.304592939980672
28	CG16833	-2.32971	0.00136555	0.720259439306979
29	CG11905	2.30719	0.0014078	0.810059794810567
30	Cyp4p2	2.29725	0.00150481	0.000685805011517976
31	Cpr67B	2.2694	0.00153205	0.313569735294304
32	Fuca	-2.21054	0.00160799	0.718030044152089
33	Cip4	2.15392	0.00161	0.410350569116489
34	CG13337	-2.1045	0.00162048	0.370785838588849
35	CG30287	-2.10244	0.00165902	0.601091585811302
36	CG1986	2.09091	0.00173978	0.757671286032475
37	sug	-2.06978	0.00179849	0.0325691442198922
38	srpk79D	-2.05707	0.00180537	0.410350569116489
39	CG6013	-2.04527	0.00190167	0.00446100132839132
40	CG12321	-2.0448	0.0019645	0.00739315515446013
41	CG5255	-2.04174	0.00200911	0.612971670754592
42	CG7556	-2.03705	0.00202923	0.757671286032475
43	CG42260	2.03106	0.00224136	0.446330297680654
44	CG15263	2.02734	0.00256942	0.201265427221489
45	RAF2	2.02461	0.0026927	0.703729977445341
46	CG30273	2.01828	0.00283577	0.655615077905348

47	CG8204	2.01675	0.00285028	0.456517577783737
48	CkIIalpha-i3	-2.00184	0.00292482	0.515272378438183
49	CG1850	1.99953	0.00295152	0.767310711680587
50	CG31053	1.98773	0.00295442	0.71310149683706
51	pim	-1.93028	0.00300947	0.345051449896687
52	CG10051	1.91159	0.00309077	0.515272378438183
53	Unc-115b	1.91002	0.00309813	0.623761030708552
54	CG13449	-1.88208	0.00345482	0.542281014973107
55	CG18853	-1.86098	0.00363686	0.000725410063268473
56	CG13332	1.85506	0.00399976	0.410350569116489
57	CG18557	-1.85285	0.00432744	0.60394231822784
58	CG9492	1.82938	0.00446582	0.638216387199714
59	ana2	-1.79887	0.00462853	0.00926829053326647
60	CG3038	-1.77446	0.00480096	0.689530427731995
61	CG42249	1.76611	0.0048966	0.60394231822784
62	Unc-115b	1.7652	0.00490697	0.515272378438183
63	CG4914	1.74158	0.00522727	0.283551836247826
64	CG40486	-1.73809	0.00542741	0.674634894888049
65	Gr5a	-1.73769	0.00552089	0.757671286032475
66	CG11052	-1.7274	0.0058479	0.0148934195083961
67	CG13058	1.72471	0.0059266	0.685444086304112
68	Osi24	-1.71825	0.006593	0.529213792133917
69	CG43255	1.71453	0.00700061	0.515272378438183
70	Cpr92F	-1.7112	0.00705661	0.768785029629205
71	Crz	1.70288	0.00706057	0.373604269292685
72	NetA	1.70161	0.00710124	0.515272378438183
73	CG34353	1.69654	0.00718051	0.510440685208423
74	Dh44-R2	1.68446	0.00739286	0.37654897980064
75	CG8785	-1.67352	0.00746657	0.757671286032475
76	CG9727	1.67237	0.00755113	0.577412115192347
77	CG8907	-1.66909	0.00760273	0.757671286032475
78	CG33156	-1.66705	0.00765374	0.152260802111618
79	CG13366	-1.65502	0.00782201	0.926674971425478
80	CG45060	1.65033	0.00823601	0.946909554600003
81	SoYb	1.6429	0.0085418	0.318050481579326
82	CG9344	-1.63518	0.00864931	0.37654897980064
83	twe	1.63176	0.00879182	0.698126015505308
84	w	-1.63166	0.00896474	0.757671286032475
85	Tsp26A	1.62643	0.00993769	0.656120442094966
86	H15	-1.62444	0.00993795	0.757671286032475
87	Cht5	1.62073	0.010016	0.19849734503522
88	CG43736	1.5981	0.0100991	0.757671286032475
89	CG33509	-1.59601	0.0101376	0.705620268150453
90	CG42272	-1.59193	0.0103613	0.185075946469654
91	Su(var)2-10	-1.59143	0.0104616	0.254401862833737
92	CG5174	-1.59058	0.0106618	0.52608014908839
93	Unc-115a	1.57537	0.0111724	0.680309356244104
94	CG11437	1.57417	0.0111973	0.478501920261311
95	CG17777	1.5678	0.0112723	0.76412651106681

96	CG18599	1.55741	0.0116148	0.460543835556973
97	CG30456	-1.54652	0.0116155	0.515272378438183
98	CG17544	1.53515	0.0116179	0.37654897980064
99	CG11127	1.52394	0.0116308	0.136407913480719
100	Shab	-1.51353	0.0119862	0.892302072347787
101	PGRP-LD	-1.5118	0.0120058	0.0575532766525622
102	RpS21	-1.50811	0.0129574	0.185075946469654
103	CG34256	1.50701	0.0129618	0.757671286032475
104	app	1.50164	0.0132478	0.685444086304112
105	CG5780	1.49944	0.013532	0.560115684285533
106	CG43117	1.49854	0.0136155	0.426975388846402
107	CG18473	-1.49322	0.0138536	0.338723935257358
108	CG6656	-1.48905	0.014108	0.402864031617179
109	CG15754	-1.48559	0.01469	0.998184828010939
110	CG9769	1.47962	0.0148129	0.597159971058699
111	CG10936	-1.4795	0.0149182	0.586440703892583
112	Obp69a	1.47424	0.0150262	0.757671286032475
113	Galphaq	-1.47412	0.0154716	0.811059173270396
114	CG15529	1.47391	0.0155806	0.708329396325653
115	CG14353	1.46901	0.0161262	0.600880148506273
116	Trpm	1.44942	0.0162739	0.757671286032475
117	CG44437	-1.44453	0.0163787	0.757671286032475
118	Mppe	-1.43716	0.0166407	0.757671286032475
119	CG12090	1.43333	0.0167062	0.60394231822784
120	scpr-C	-1.4309	0.0167954	0.185075946469654
121	CG18609	1.42978	0.0168238	0.757671286032475
122	Cf2	1.41871	0.0172977	0.478501920261311
123	CG10881	1.41845	0.0176172	0.785227515051893
124	CG13071	-1.41524	0.017657	0.757671286032475
125	CG11052	-1.41391	0.0177563	0.136407913480719
126	CG3165	1.41221	0.0177836	0.866963580572175
127	Ubx	1.41099	0.0180197	0.757671286032475
128	Drs	-1.41071	0.0182089	0.812456898699683
129	CG30345	-1.40841	0.0182174	0.704738264328017
130	CG31415	1.40269	0.0189974	0.667850335932463
131	spir	1.39872	0.0190942	0.318714911393125
132	CG3857	1.39422	0.0192596	0.474792604224839
133	CG11453	1.38702	0.0195767	0.698126015505308
134	CG12645	-1.38344	0.019582	0.753699458372164
135	abd-A	-1.38168	0.0197068	0.761569163840286
136	mthl8	-1.38128	0.0198655	0.757671286032475
137	Cpr5C	1.37575	0.020071	0.832213572731741
138	BCAS2	-1.37207	0.0202448	0.612971670754592
139	CG12885	-1.3681	0.02031	0.515272378438183
140	CG43391	-1.36349	0.0206396	0.889505762045968
141	Drsl5	1.36343	0.0207416	0.718030044152089
142	CG15728	-1.35969	0.0208127	0.125877324937865
143	CG6432	-1.34716	0.0210749	0.757671286032475
144	RhoGAP100F	1.34606	0.0213545	0.757671286032475

145	ppk5	1.34476	0.021929	0.337322528858992
146	CG9676	1.34457	0.0219301	0.757671286032475
147	E(Pc)	1.34122	0.0220582	0.757671286032475
148	Ank2	1.33731	0.0220951	0.97897872806019
149	CG11069	-1.33723	0.0221131	0.655615077905348
150	CG9150	1.33489	0.0222569	0.337096483355174
151	CG7458	1.33339	0.0224691	0.757671286032475
152	vimar	-1.33174	0.0226399	0.757671286032475
153	CG43894	1.32797	0.022762	0.742527561447521
154	2mit	-1.32023	0.0229231	0.757671286032475
155	cry	1.31883	0.023081	0.402864031617179
156	Gr94a	1.31855	0.023118	0.79422226448489
157	Cpr49Af	1.3161	0.0234645	0.698126015505308
158	CG14511	-1.31468	0.023544	0.103035609686662
159	CG13890	1.30567	0.0236862	0.757671286032475
160	Rel	-1.30107	0.0238607	0.757671286032475
161	l(2)efl	-1.293	0.0240444	0.704738264328017
162	CG8414	1.29291	0.024168	0.653197880960586
163	Ser	-1.29045	0.0245358	0.656120442094966
164	fand	-1.28856	0.024746	0.757671286032475
165	pre-mod(mdg4)-B	1.28848	0.0247947	0.185075946469654
166	CG34215	-1.28684	0.0250431	0.0917098158385881
167	Sik2	-1.2768	0.0251667	0.757671286032475
168	CG11563	1.27595	0.0252203	0.60394231822784
169	Ir92a	1.2757	0.0252352	0.674634894888049
170	Spn77Bc	-1.27436	0.0252821	0.0031971724352473
171	CG8925	-1.27163	0.0255192	0.446330297680654
172	MsR2	1.26933	0.025872	0.703729977445341
173	adat	1.26848	0.0261556	0.60394231822784
174	Oseg6	-1.26807	0.0267948	0.560702669736188
175	TpnC47D	1.26508	0.0268371	0.980473697520996
176	CG4998	-1.25891	0.0268406	0.646131472652424
177	Roc1b	1.25189	0.0269176	0.869247934884916
178	ppk31	1.2494	0.0270496	0.764613992848028
179	CG5953	-1.24839	0.0273968	0.674634894888049
180	G6P	1.24727	0.027399	0.656120442094966
181	CG34178	1.24395	0.0274693	0.620203280699957
182	Alg10	-1.24353	0.02754	0.664008343570246
183	ey	-1.24188	0.0276617	0.754345163817909
184	fra	-1.24008	0.0278938	0.60394231822784
185	CG43689	1.23984	0.0279686	0.949117163038273
186	Vmat	1.23964	0.0281828	0.698126015505308
187	Efhc1.2	-1.23805	0.028381	0.757671286032475
188	hbs	-1.23377	0.0285023	0.757671286032475
189	stum	-1.23053	0.0295451	0.757671286032475
190	CG31266	1.22864	0.0296944	0.754345163817909
191	Cyp305a1	1.22651	0.0297107	0.300944229830809
192	NimA	1.22579	0.0298256	0.541778667392807
193	Corp	1.22439	0.0298997	0.708329396325653

194	CG44815	-1.21837	0.0299521	0.416187456435379
195	oys	1.21657	0.0301115	0.873359308855355
196	CG17378	1.21617	0.0303113	0.757671286032475
197	Cp7Fb	-1.21031	0.0305952	0.757671286032475
198	CG2698	-1.20909	0.0306175	0.757671286032475
199	how	1.20882	0.0306534	0.757671286032475
200	ntc	-1.20613	0.0306794	0.757671286032475
201	CG9317	1.20566	0.03068	0.870613938961838
202	CG6347	-1.20173	0.0307637	0.474005157674284
203	CG12826	-1.20156	0.0308084	0.757671286032475
204	CG13742	1.19826	0.0312148	0.820199984709553
205	CG43164	1.19513	0.0314291	0.858480282290443
206	CG10764	-1.19312	0.0319201	0.764613992848028
207	CG31776	-1.19183	0.0323258	0.768065918030128
208	kkv	-1.1914	0.0324095	0.795569015924202
209	Ugt58Fa	-1.19133	0.0325166	0.544319570414765
210	CG42494	1.19056	0.0325713	0.646131472652424
211	CG15186	1.18856	0.0325867	0.729344169809764
212	CG30378	1.18486	0.0329033	0.779909886089331
213	CG3927	-1.1847	0.0329359	0.757671286032475
214	CG2926	1.18463	0.0331619	0.757671286032475
215	CG3611	1.17806	0.0331996	0.757671286032475
216	trp	-1.17215	0.0335682	0.977543083444176
217	CG2051	1.17096	0.0336357	0.85310059450542
218	NK7.1	-1.16671	0.0337179	0.345051449896687
219	CG30354	1.16302	0.0337396	0.757671286032475
220	gb	1.16268	0.0339097	0.757671286032475
221	CG15547	1.16248	0.0340768	0.754345163817909
222	CG14669	-1.15606	0.0341479	0.757671286032475
223	ATPCL	-1.15597	0.0344406	0.217003150926687
224	CG12239	-1.15528	0.0349165	0.893214191740644
225	Cyp12b2	1.14402	0.035066	0.818458566098791
226	pyd3	-1.14232	0.0354067	0.217003150926687
227	CG30291	1.13691	0.0355614	0.583946139978677
228	Mf	-1.13655	0.0355717	0.18242327423704
229	CG13871	-1.13605	0.0356	0.757671286032475
230	Ndae1	1.13523	0.0356401	0.757671286032475
231	Graf	-1.12934	0.0362437	0.757671286032475
232	SP2637	1.12512	0.0369299	0.515272378438183
233	PsGEF	1.11993	0.0370435	0.757671286032475
234	Mbs	-1.11936	0.0370757	0.117771822149298
235	CG6094	-1.11616	0.0375951	0.338723935257358
236	CG32249	-1.11404	0.0380092	0.698126015505308
237	CG12688	-1.11331	0.038081	0.757671286032475
238	CG32548	-1.11257	0.0380958	0.565141704459875
239	CG15864	-1.1118	0.0384788	0.757671286032475
240	CAH1	1.10843	0.0385398	0.732551907716915
241	hb	1.10681	0.0387301	0.760978857472456
242	CG34279	1.10667	0.0387928	0.757671286032475

243	mwh	1.10515	0.0389275	0.805316630563153
244	CG8343	1.10358	0.0392012	0.757671286032475
245	Vsx2	-1.09938	0.0398728	0.689202729169586
246	Men	-1.099	0.0401556	0.419316641790998
247	CG42869	-1.0948	0.0411719	0.754345163817909
248	RnpS1	-1.0929	0.0414748	0.757671286032475
249	CG11160	-1.09222	0.0416331	0.789101253661469
250	CG10639	1.09216	0.0418079	0.902346672483455
251	Lsd-1	-1.09109	0.0424437	0.217003150926687
252	CG44956	-1.09055	0.0425099	0.757671286032475
253	CG43816	-1.0885	0.0425149	0.757671286032475
254	Galphaq	1.08808	0.0425892	0.796295318788887
255	CG4495	-1.0879	0.0427443	0.757671286032475
256	CG13954	-1.08412	0.0432526	0.757671286032475
257	Cyp9h1	-1.08047	0.043382	0.217003150926687
258	klar	-1.08045	0.0434005	0.757671286032475
259	dap	1.07654	0.0437822	0.658507941537216
260	Rh5	1.07652	0.0438728	0.861522766741487
261	Ppcs	-1.07436	0.0439704	0.757671286032475
262	CG3437	1.07394	0.0440457	0.757671286032475
263	Ykt6	-1.06788	0.0441035	0.580288475835106
264	Nmda1	-1.06676	0.0441248	0.812456898699683
265	NimC1	1.06644	0.0443398	0.757671286032475
266	CG8613	1.06201	0.0445549	0.757671286032475
267	ZnT77C	-1.05884	0.0449808	0.716215400866489
268	CG12278	-1.05826	0.0450065	0.762985353777346
269	CG9953	1.05689	0.0451089	0.674634894888049
270	Cry	-1.05666	0.0451426	0.963389669809287
271	CG32563	-1.05287	0.0452034	0.757671286032475
272	CG30460	1.05162	0.0453045	0.757671286032475
273	CG6439	1.05127	0.0454615	0.757671286032475
274	CG17571	-1.04771	0.0457344	0.789101253661469
275	Samuel	-1.04698	0.0459594	0.757671286032475
276	Fbp2	-1.04694	0.0460628	0.811059173270396
277	Diedel	1.0453	0.0462227	0.720259439306979
278	CG44158	-1.04112	0.0465312	0.761444923782125
279	lectin-37Da	-1.04055	0.046746	0.689202729169586
280	CG15140	-1.04048	0.0467607	0.757671286032475
281	CG12034	1.03684	0.0469281	0.757671286032475
282	CG18265	-1.0352	0.0469306	0.757671286032475
283	CG40178	1.03277	0.0470354	0.701992317213399
284	CG4788	-1.03084	0.0471293	0.676907519574074
285	lush	-1.03039	0.0471429	0.944056072773374
286	Ddr	1.02787	0.047353	0.757671286032475
287	5-HT2B	-1.02777	0.0474312	0.757671286032475
288	CG32413	1.02726	0.0474663	0.877331179953365
289	Atg6	-1.02492	0.0480023	0.718030044152089
290	CG15169	-1.0239	0.0480974	0.780281013130785
291	Send1	1.01948	0.0481713	0.790133715398166

292	CG6852	-1.01574	0.0486655	0.419117007806657
293	CG44252	1.01486	0.048681	0.658507941537216
294	rdgB	-1.01157	0.0490754	0.869585043068839
295	dlg1	-1.00989	0.0491913	0.962205003241268
296	Ced-12	1.00967	0.0493947	0.757671286032475
297	Mhcl	1.00795	0.0497068	0.808129121298945
298	sls	-1.00626	0.0497349	0.757671286032475

#

Figure S7: List of genes from the heatmap B (Fig. 6), with their fold change, Welch's t-test p-values and ANOVA p-values adjusted for FDR.

#	gene names	fold change	Welch's t-test p-value	adjusted p-value
1	Sfp77F	6.51129	3.831e-06	0.0203261123926112
2	CG44434	5.90493	1.1188e-05	0.117771822149298
3	CG17597	5.84949	1.439e-05	0.00080508688024098
4	CG18853	-5.27306	2.1299e-05	0.000725410063268473
5	l(2)01289	4.86974	4.4686e-05	0.338723935257358
6	CG4962	4.7607	7.3697e-05	0.00319853323991776
7	Spn77Bc	4.12358	8.1678e-05	0.0031971724352473
8	CG10116	3.98518	8.6885e-05	1.27825724798632e-05
9	Prx2540-2	3.8214	0.000155382	0.0351548152413644
10	CG4914	3.76188	0.000191408	0.283551836247826
11	ppk5	3.7145	0.000229909	0.337322528858992
12	CG45050	3.57546	0.000278517	0.949361446541045
13	MED23	3.53791	0.000331093	0.103035609686662
14	CG45307	3.49958	0.000342575	0.794327317828636
15	CG5379	3.44788	0.000367929	0.373604269292685
16	didum	3.43892	0.000370379	0.0203261123926112
17	CG13796	3.29946	0.000475332	0.00739315515446013
18	CG7724	3.27205	0.000491076	0.493039848347587
19	Osi6	3.17786	0.000568794	0.757671286032475
20	scpr-B	-3.16498	0.000636626	0.000137232337333212
21	CG34215	3.14637	0.000656584	0.0917098158385881
22	CG17119	-3.11966	0.000674874	0.444893326850019
23	Spn77Bb	3.09705	0.00071384	0.00740922120863881
24	spn-E	-3.03927	0.000771868	0.69690275476614
25	Drsl6	3.03514	0.000781717	0.757671286032475
26	moody	3.00668	0.000786146	0.318714911393125
27	Prx3	2.96576	0.000823709	0.0130535106540621
28	CG42825	2.95564	0.000844032	0.337322528858992
29	a5	2.87848	0.000892628	0.794327317828636
30	KdelR	-2.82735	0.00125891	0.15679178778124
31	Thor	2.77886	0.00127305	0.544319570414765
32	Or49b	2.64307	0.00128544	0.577412115192347
33	Fak	2.63672	0.00135442	0.757671286032475
34	Cyp4p2	2.5753	0.00178069	0.000685805011517976
35	CG8051	-2.4897	0.00188382	0.757671286032475
36	Ir68a	2.43738	0.00202126	0.757671286032475
37	CG10332	2.4183	0.00204866	0.656120442094966
38	ana2	-2.41459	0.00210285	0.00926829053326647
39	Muc18B	2.41113	0.00218362	0.233334183763622
40	CG15695	2.38598	0.00259846	0.603462899825576
41	Ucrh	-2.34928	0.00265089	0.789101253661469
42	nord	2.34903	0.0028089	0.757671286032475
43	Yp1	2.30583	0.00288609	0.757671286032475
44	Aph-4	2.30292	0.00293724	0.00106010354186678
45	CG30271	2.26917	0.00296098	0.757671286032475

47	beat-IIb	2.25148	0.00308683	0.718030044152089
48	CG12038	-2.23311	0.00323145	0.337096483355174
49	AttC	2.22231	0.00352616	0.757671286032475
50	CCHa1	2.19141	0.00359099	0.882932887646482
51	CecB	2.1638	0.00371096	0.757671286032475
52	CG14511	2.12148	0.00372453	0.103035609686662
53	CG6654	2.11354	0.00376165	0.936060486692145
54	CG32425	2.10443	0.00388463	0.035630434460939
55	Cyp9h1	2.10277	0.00397957	0.217003150926687
56	pre-mod(mdg4)-T	2.09993	0.00402501	0.937691194606184
57	AdoR	2.08613	0.00429804	0.757671286032475
58	Sse	2.06361	0.00433112	0.60394231822784
59	Mf	2.05903	0.00454138	0.318050481579326
60	aurB	2.05024	0.00477322	0.00319853323991776
61	glob3	2.02889	0.00511981	0.612971670754592
62	CG43143	-1.96148	0.00513162	0.176305081634661
63	CG42388	1.95682	0.00522292	0.700217571149591
64	CG30356	-1.9528	0.00551986	0.720259439306979
65	CG5194	1.93755	0.00562964	0.656120442094966
66	TyrRII	-1.9249	0.00577886	0.656120442094966
67	CG12239	1.88827	0.00607317	0.893214191740644
68	Npc2f	-1.8676	0.0060773	0.757671286032475
69	CG14340	-1.85492	0.0063112	0.987892157887495
70	OS9	1.85058	0.00656284	0.79998400407722
71	Sema-2a	-1.79497	0.00656751	0.825365361144409
72	squ	-1.79449	0.00657358	0.708329396325653
73	CG5196	-1.77966	0.00663683	0.639974398895424
74	CG8568	1.77076	0.00705798	0.767220675485777
75	CG43175	1.76653	0.00717397	0.971110216247572
76	Obp84a	1.75253	0.0073186	0.966580039235872
77	CG42235	1.75124	0.00738197	0.757671286032475
78	Crg-1	1.74911	0.00765557	0.757671286032475
79	CG15879	1.74801	0.00797604	0.515272378438183
80	CG12768	1.74722	0.00834651	0.839701153723415
81	LKR	1.73167	0.00862928	0.464903820037865
82	Pepck	1.72918	0.0086558	0.185075946469654
83	CG43061	-1.71788	0.00899548	0.557939889828493
84	CG17777	-1.71456	0.00912279	0.76412651106681
85	Gnmt	1.71275	0.00914531	0.612971670754592
86	MESR3	1.70663	0.00923832	0.515272378438183
87	CG6125	1.70374	0.00925875	0.560702669736188
88	CG13042	-1.69994	0.00931468	0.656120442094966
89	CG6201	1.69068	0.00947101	0.786343133194672
90	Cyp309a2	1.67655	0.009692	0.60611524185225
91	CG33777	1.66759	0.00974327	0.882932887646482
92	CG45061	1.65994	0.0100667	0.757671286032475
93	Obp28a	1.65785	0.0103039	0.81450872972759
94	Mbs	-1.64687	0.0107257	0.117771822149298
95	Lkr	-1.6467	0.0109997	0.958994696950357

96	Tsp42Er	-1.62891	0.0110559	0.583946139978677
97	RpS21	1.62194	0.0111378	0.185075946469654
98	cas	1.61143	0.0111434	0.914014810661854
99	CG18446	-1.61057	0.0113296	0.786998308005735
100	CG42656	-1.6015	0.0113915	0.919974649072763
101	Ccp84Ae	1.59513	0.0115985	0.703729977445341
102	lola	1.59039	0.0123417	0.757671286032475
103	Jon99Ciii	1.58609	0.0126788	0.920548990724541
104	ck	1.58421	0.0126884	0.515272378438183
105	CG6465	1.57548	0.0127437	0.847748453162316
106	CG11498	1.57369	0.0127594	0.869186158362766
107	CG14787	-1.56451	0.0128831	0.757671286032475
108	tin	1.52277	0.013121	0.808129121298945
109	CG11585	1.51827	0.0132966	0.399910787044423
110	CecC	1.51322	0.0133601	0.98262196110908
111	Dpt	1.51298	0.0134677	0.779130109934133
112	Cht5	1.51205	0.0135004	0.19849734503522
113	Incenp	1.49696	0.0137003	0.992085055173606
114	CG16965	1.49647	0.013728	0.517581931519484
115	CG5493	-1.49411	0.0138136	0.736701755136478
116	CG9416	1.48933	0.0138881	0.513810489724607
117	CG13705	1.48496	0.0139055	0.753858363553586
118	CG31068	1.4834	0.0139157	0.676907519574074
119	CG13073	1.48319	0.0139175	0.737692593583628
120	cid	1.48002	0.0140533	0.757671286032475
121	CG18170	1.47913	0.0150815	0.767220675485777
122	AttA	1.4771	0.0151952	0.762985353777346
123	CG10131	1.46825	0.0152234	0.757671286032475
124	CG1910	-1.46405	0.0152667	0.510440685208423
125	CecA1	1.46215	0.0153075	0.768412978157496
126	Yp2	1.46159	0.0153949	0.757671286032475
127	CG42678	-1.46043	0.0155934	0.757671286032475
128	CG11236	1.45587	0.0156312	0.703729977445341
129	d	1.45359	0.0158126	0.757671286032475
130	CG43069	1.43994	0.0160138	0.757671286032475
131	a10	1.43734	0.016082	0.986920531744917
132	Sk1	1.43508	0.0161457	0.639774743709038
133	fd64A	1.42958	0.0164189	0.76412651106681
134	dpr3	-1.42625	0.0166793	0.847694448472838
135	PGRP-SC2	1.41566	0.0169905	0.757671286032475
136	CG15263	1.41268	0.0174424	0.201265427221489
137	CG13931	1.40505	0.0178838	0.789101253661469
138	sug	1.40332	0.0181763	0.0325691442198922
139	ade3	1.38869	0.0185194	0.515272378438183
140	Tsp2A	1.37965	0.0185949	0.864125821276335
141	GNBP-like3	-1.37657	0.0186785	0.757671286032475
142	CG42299	1.36938	0.0189309	0.631468894385972
143	AttB	1.36844	0.0189498	0.870148320789815
144	AcCoAS	-1.36838	0.0191718	0.560702669736188

145	CG43236	1.36634	0.0197726	0.757671286032475
146	CG18249	1.36134	0.0198078	0.757671286032475
147	dgt3	1.36008	0.0201058	0.52608014908839
148	Vkor	1.33822	0.0202211	0.757671286032475
149	mus308	1.32178	0.0202996	0.881824933241346
150	Ugt37b1	-1.3213	0.0204572	0.631468894385972
151	CG5849	1.31736	0.0206084	0.757671286032475
152	CG34223	1.31635	0.020718	0.689202729169586
153	CG11052	1.30606	0.0214657	0.0148934195083961
154	Wnt4	1.30217	0.0216272	0.784326431234337
155	VhaAC39-2	1.29747	0.0218466	0.597159971058699
156	GstD2	1.29119	0.0220167	0.714200430369868
157	Mdr50	1.29018	0.0220913	0.97569307778491
158	Crz	1.28767	0.0223014	0.373604269292685
159	Cyp6a14	1.2876	0.0228357	0.238672048089959
160	Mf	1.28692	0.0230659	0.18242327423704
161	CG41562	1.28507	0.0231759	0.370785838588849
162	CG30091	1.28103	0.0234816	0.754345163817909
163	AttD	1.27234	0.0235813	0.79097745891082
164	ppk22	1.26813	0.0236832	0.513810489724607
165	Lsp1alpha	1.26502	0.0240206	0.62800356883976
166	Gk	-1.25994	0.0242333	0.757671286032475
167	CG4880	1.25911	0.0245833	0.830244354389019
168	CG11127	1.25415	0.0246681	0.136407913480719
169	CG17104	1.25379	0.0247171	0.757671286032475
170	CG9629	1.24511	0.0247481	0.757671286032475
171	CG14109	1.23553	0.0254876	0.518207910131528
172	CG11425	-1.23276	0.0255326	0.76207521973134
173	CG31606	-1.23189	0.0260837	0.60394231822784
174	CG11391	1.22958	0.0262759	0.780645969120174
175	Tsp42En	1.21825	0.02829	0.787254624472035
176	Lsd-1	-1.21138	0.0285809	0.217003150926687
177	Eip78C	-1.20835	0.0286659	0.757671286032475
178	CG32532	1.20395	0.0286693	0.757671286032475
179	St1	-1.20287	0.028715	0.757671286032475
180	CG42464	1.20207	0.0289578	0.793455514327327
181	dob	-1.20106	0.0290286	0.698126015505308
182	MtnD	1.1995	0.0291606	0.689202729169586
183	CG15096	1.19475	0.030324	0.553526074607077
184	CG7059	1.19366	0.030748	0.583946139978677
185	Dop1R2	1.19146	0.0307558	0.79998400407722
186	qkr58E-1	1.19033	0.0311344	0.861483049534217
187	CG16837	1.18944	0.0312479	0.703729977445341
188	CG34112	1.18921	0.031396	0.657320795647897
189	Obp99b	1.18716	0.0321533	0.560702669736188
190	CG32713	1.18503	0.03273	0.757671286032475
191	CG5399	1.18476	0.0327728	0.676874969229701
192	tld	-1.18245	0.0327844	0.757671286032475
193	Akap200	-1.18189	0.0330219	0.322051422915093

194	Sardh	1.18087	0.0331878	0.784326431234337
195	Oamb	1.17821	0.0334776	0.894447324200704
196	Obp19b	1.16555	0.0336718	0.757671286032475
197	Acp63F	1.15951	0.0337052	0.895944951752564
198	Su(var)2-10	-1.15649	0.0343421	0.254401862833737
199	Pdk1	1.15623	0.0344688	0.577412115192347
200	Act79B	1.1513	0.0347214	0.656120442094966
201	CG10993	1.15012	0.0350201	0.635544830390417
202	Spc25	-1.14595	0.0359678	0.79422226448489
203	CCHa2	1.14404	0.0363208	0.757671286032475
204	CG14075	1.14032	0.0363572	0.757671286032475
205	LysX	1.13853	0.0363603	0.757671286032475
206	CG33099	-1.13758	0.0365044	0.757671286032475
207	Os-C	-1.13442	0.036739	0.841400996480479
208	CG6503	1.13101	0.036851	0.757671286032475
209	CG3604	1.13034	0.037024	0.760614077583408
210	CG30424	1.12904	0.0372237	0.900104125045228
211	CG9452	1.12575	0.0373787	0.995407658498039
212	CG18003	1.1254	0.0382828	0.786476232588256
213	GILT3	-1.12463	0.038649	0.884879634636984
214	p38c	-1.12182	0.0388603	0.757671286032475
215	CG6415	-1.11668	0.0389598	0.757671286032475
216	Dro	1.11574	0.0391724	0.79656208149081
217	Jhe	1.11468	0.0391784	0.757671286032475
218	CG3397	-1.11008	0.0391861	0.757671286032475
219	CG18473	1.10984	0.0393432	0.338723935257358
220	CG3408	1.10554	0.0394621	0.742527561447521
221	CG3699	1.10473	0.039598	0.706395798574486
222	CG14332	-1.09994	0.0400104	0.563486344269827
223	CG7997	-1.09451	0.0400372	0.656120442094966
224	Lsp2	1.09335	0.040177	0.757671286032475
225	CG31370	-1.09187	0.0406683	0.757671286032475
226	CG10184	-1.0855	0.0407747	0.577412115192347
227	ade3	-1.08343	0.041491	0.742900535529728
228	inaF-C	1.08097	0.0415482	0.597159971058699
229	Ics	1.07858	0.0418484	0.79097745891082
230	Tsp42Eo	-1.0765	0.0421361	0.767310711680587
231	Acox57D-d	1.07446	0.0421741	0.338723935257358
232	S6k	1.07324	0.0426941	0.477779030081756
233	CG9747	1.07199	0.0428602	0.689202729169586
234	CG13741	1.07019	0.0437711	0.757671286032475
235	Fmo-2	-1.0648	0.0438563	0.757671286032475
236	fng	-1.06247	0.0440686	0.759159458333388
237	CG14630	1.05976	0.0445504	0.757671286032475
238	Sodh-2	1.05567	0.04477	0.577412115192347
239	CG5023	-1.05095	0.0449714	0.757671286032475
240	Obp69a	1.04763	0.0450221	0.757671286032475
241	CG9657	-1.04755	0.0452221	0.757671286032475
242	CG17572	-1.04499	0.0454333	0.64941329848826

243	CG4408	1.04376	0.0456842	0.720807556416846
244	Dbi	-1.04105	0.045898	0.757671286032475
245	Obp83b	1.04007	0.0459627	0.907135834632983
246	Amy-p	1.03934	0.0461889	0.757671286032475
247	CCAP	1.03875	0.0462793	0.757671286032475
248	CG7341	1.03135	0.0468197	0.757671286032475
249	CG14688	-1.0308	0.046899	0.757671286032475
250	Lsp1beta	1.03005	0.0470491	0.701992317213399
251	CG11674	1.0298	0.0474124	0.79097745891082
252	Cyp4e3	-1.02871	0.0474493	0.757671286032475
253	CG13085	-1.02729	0.0476851	0.612971670754592
254	CG11889	1.02267	0.0477073	0.874319410533089
255	Proc-R	1.01973	0.0478198	0.715144154831622
256	lin-28	1.01827	0.0479084	0.819455056999577
257	CG3735	1.01785	0.0483671	0.513810489724607
258	tobi	-1.01756	0.0484321	0.757671286032475
259	btl	-1.01701	0.0484871	0.76412651106681
260	Nlg1	1.01268	0.0488513	0.337096483355174
261	CG10512	1.01048	0.0495486	0.787254624472035
262	CG12998	-1.00676	0.0496647	0.757671286032475
263	CG13840	-1.00429	0.0497395	0.757671286032475

#
